# Comprehensive analysis of 33 human cancers reveals clinical implications and immunotherapeutic value of the solute carrier family 35 member A2

**DOI:** 10.3389/fimmu.2023.1155182

**Published:** 2023-05-18

**Authors:** Shengshan Xu, Xiguang Chen, Jianxiong Fang, Hongyu Chu, Shuo Fang, Leli Zeng, Hansu Ma, Tianzhi Zhang, Yu Chen, Tao Wang, Xin Zhang, Tao Shen, Youbin Zheng, Dongming Xu, Zhuming Lu, Yihang Pan, Yuchen Liu

**Affiliations:** ^1^ Department of Thoracic Surgery, Jiangmen Central Hospital, Jiangmen, Guangdong, China; ^2^ Scientific Research Center, The Seventh Affiliated Hospital, Sun Yat-Sen University, Shenzhen, Guangdong, China; ^3^ Department of Medical Oncology, The First Affiliated Hospital of University of South China, Hengyang, Hunan, China; ^4^ Department of Urology, Jiangmen Central Hospital, Jiangmen, Guangdong, China; ^5^ Department of Gastrointestinal, Colorectal and Anal Surgery, China-Japan Union Hospital of Jilin University, Changchun, China; ^6^ Department of Oncology, The Seventh Affiliated Hospital, Sun Yat-Sen University, Shenzhen, Guangdong, China; ^7^ Department of Pathology, The Seventh Affiliated Hospital of Sun Yat-Sen University, Shenzhen, China; ^8^ Clinical Experimental Center, Jiangmen Key Laboratory of Clinical Biobanks and Translational Research, Jiangmen Central Hospital, Jiangmen, China; ^9^ Department of Radiology, Jiangmen Wuyi Hospital of Traditional Chinese Medicine, Jiangmen, Guangdong, China; ^10^ Department of Neurosurgery, The County Hospital of Qianguo, Songyuan, Jilin, China

**Keywords:** pan-cancer analysis, glycosylation, SLC35A2, tumor microenvironment, immunotherapy

## Abstract

**Background:**

Solute carrier family 35 member A2 (SLC35A2), which belongs to the SLC35 solute carrier family of human nucleoside sugar transporters, has shown regulatory roles in various tumors and neoplasms. However, the function of SLC35A2 across human cancers remains to be systematically assessed. Insights into the prediction ability of SLC35A2 in clinical practice and immunotherapy response remains limited.

**Materials and methods:**

We obtained the gene expression and protein levels of SLC35A2 in a variety of tumors from Molecular Taxonomy of Breast Cancer International Consortium, The Cancer Genome Atlas, Gene Expression Omnibus, Chinese Glioma Genome Atlas, and Human Protein Atlas databases. The SLC35A2 level was validated by immunohistochemistry. The predictive value for prognosis was evaluated by Kaplan–Meier survival and Cox regression analyses. Correlations between SLC35A2 expression and DNA methylation, genetic alterations, tumor mutation burden (TMB), microsatellite instability (MSI), and tumor microenvironment were performed using Spearman’s correlation analysis. The possible downstream pathways of SLC35A2 in different human cancers were explored using gene set variation analysis. The potential role of SLC35A2 in the tumor immune microenvironment was evaluated *via* EPIC, CIBERSORT, MCP-counter, CIBERSORT-ABS, quanTIseq, TIMER, and xCell algorithms. The difference in the immunotherapeutic response of SLC35A2 under different expression conditions was evaluated by the tumor immune dysfunction and exclusion (TIDE) score as well as four independent immunotherapy cohorts, which includes patients with bladder urothelial carcinoma (BLCA, N = 299), non–small cell lung cancer (NSCLC, N = 72 and N = 36) and skin cutaneous melanoma (SKCM, N = 25). Potential drugs were identified using the CellMiner database and molecular docking.

**Results:**

SLC35A2 exhibited abnormally high or low expression in 23 cancers and was significantly associated with the prognosis. In various cancers, SLC35A2 expression and mammalian target of rapamycin complex 1 signaling were positively correlated. Multiple algorithmic immune infiltration analyses suggested an inverse relation between SLC35A2 expression and infiltrating immune cells, which includes CD4+T cells, CD8+T cells, B cells, and natural killer cells (NK) in various tumors. Furthermore, SLC35A2 expression was significantly correlated with pan-cancer immune checkpoints, TMB, MSI, and TIDE genes. SLC35A2 showed significant predictive value for the immunotherapy response of patients with diverse cancers. Two drugs, vismodegib and abiraterone, were identified, and the free binding energy of cytochrome P17 with abiraterone was higher than that of SLC35A2 with abiraterone.

**Conclusion:**

Our study revealed that SLC35A2 is upregulated in 20 types of cancer, including lung adenocarcinoma (LUAD), breast invasive carcinoma (BRCA), colon adenocarcinoma (COAD), and lung squamous cell carcinoma (LUSC). The upregulated SLC35A2 in five cancer types indicates a poor prognosis. Furthermore, there was a positive correlation between the overexpression of SLC35A2 and reduced lymphocyte infiltration in 13 cancer types, including BRCA and COAD. Based on data from several clinical trials, patients with LUAD, LUSC, SKCM, and BLCA who exhibited high SLC35A2 expression may experience improved immunotherapy response. Therefore, SLC35A2 could be considered a potential predictive biomarker for the prognosis and immunotherapy efficacy of various tumors. Our study provides a theoretical basis for further investigating its prognostic and therapeutic potentials.

## 1 Introduction

Recent studies have revealed that aberrant glycosylation contributes to the key pathological steps of cancer development (1). Aberrant glycosylation also exhibits definite effects on specific cellular functions, serving as a biomarker in multiple tumor types: aberrant glycosylation of cancer antigen (CA)125 leads to cancer progression, CD43 contributes to metastasis, CA19-9 contributes to tumor recurrence, and CD147 contributes to drug resistance (2–5). Furthermore, aberrant glycosylation in cancers might generate neoantigens and affect glycan-binding receptors. Aberrant glycosylation promotes the phenotypic conversion of various tumors to inflammatory phenotypes and facilitates the shaping of the immune microenvironment along with regulating immunotherapy response (6). N-glycosylation has been reported to be crucial for maintaining the structural stability of PD-1, thereby enhancing its immunosuppressive activity against T cells (7). Therefore, evaluating aberrant glycosylation in cancer might provide insights regarding potential targets for predicting prognosis and developing personalized cancer treatments.

Based on the significance of glycosylation, we previously identified a high-risk gene named solute carrier family 35 member A2 (SLC35A2) and developed a novel risk stratification signature. SLC35A2 is a human nucleoside sugar transporter and encodes a multichannel membrane protein that affects the trafficking of uridine diphosphate-galactose to Golgi vesicles. In the production of glycans, SLC35A2 is the glycosyl donor in Golgi vesicles (8). SLC35A2 is also critical for the synthesis of galactosylceramide and galactosyl diglycerides in the endoplasmic reticulum (9). With regard to tumorigenesis, increased SLC35A2 levels have been correlated with a worse prognosis in hormone receptor-positive breast cancer (10). Similarly, SLC35A2 regulates cellular glycosylation in hepatocellular carcinoma metastasis (11). He et al. and Kotolloshi et al. identified that SLC35F2 is essential for papillary thyroid carcinoma and bladder cancer progression (12, 13). These findings suggest that SLC35A2 is a crucial contributor to malignant phenotypes. However, the prognostic or predictive values of SLC35A2 on immunotherapies have not yet been reported in multiple cancer types. Therefore, we performed a pan-cancer analysis of SLC35A2 and found that SLC35A2 is a promising indicator of prognosis and immunotherapy response.

## 2 Materials and methods

### 2.1 Data sources and processing

We downloaded the pan-cancer gene expression data and corresponding clinical information from the Cancer Genome Atlas (TCGA, http://cancergenome.nih.gov/), the Molecular Taxonomy of Breast Cancer International Consortium (METABRIC, http://www.cbioportal.org), the Chinese Glioma Genome Atlas (CGGA, http://www.cgga.org.cn) databases. **Supplementary Table 1** shows the abbreviations and number of samples for 33 cancers.

The datasets including gene expression profiles and corresponding clinical information of GSE69053, GSE53625, GSE13507, GSE65858, GSE2748, GSE37642, GSE144269, GSE30219, GSE157009, GSE17118, GSE32062, GSE97211, GSE17674, GSE194234, and GSE119041 were downloaded from the Gene Expression Omnibus database (GEO, https://www.ncbi.nlm.nih.gov/geo/). The immunotherapy response datasets were obtained from GEO (GSE35640, GSE61676, and GSE78220). These datasets are shown in **Supplementary Table 2**. The somatic mutation data were collected from the UCSC Xena database (http://xena.ucsc.edu/).

### 2.2 Genetic alteration analysis

We used the COSMIC (https://cancer.sanger.ac.uk/cosmic/) and the cBioPortal (http://cbioportal.org) databases to explore the mutation types and the distribution of SLC35A2 mutational differences across human cancers, respectively. The GSCA website (http://bioinfo.life.hust.edu.cn/GSCA/#/) was performed to explore the relevance between the methylation, copy number variation (CNV) and SLC35A2 expression. We identify the relationship between survival prognosis and SLC35A2 methylation *via* UALCAN (http://ualcan.path.uab.edu/) and DNMIVD databases (http://119.3.41.228/dnmivd/). The association between SLC35A2 mutations and gene expression was explored through Tumor immune estimation resource version 2 (TIMER2, http://timer.cistrome.org/).

### 2.3 Gene expression analysis

We accessed the expression of SLC35A2 across different cell and tissue types using the Human Protein Atlas database (HPA, https://www.proteinatlas.org). Next, TIMER2 was conducted to investigate the pan-cancer expression of SLC35A2. The expression of SLC35A2 in tumors and adjacent normal tissues was gathered from the genotype-tissue expression (GTEx) database with respect to cancer types lacking healthy samples or those with less than two healthy samples. We then conducted a meta-analysis of “tumor vs. normal” SLC35A2 expression in non-small cell lung cancer using the LUNG CANCER EXPLORER (https://lce.biohpc.swmed.edu/lungcancer/) service. We used the UALCAN web service to identify the differential SLC35A2 protein levels in liver hepatocellular carcinoma (LIHC), breast invasive carcinoma (BRCA), and glioblastoma multiforme (GBM). Subsequently, we used the HPA database to observe the differences in immunohistochemical (IHC) images of SLC35A2 levels between tumor and corresponding non-neoplastic tissues.

### 2.4 Tissue microarray and IHC analyses

Lung cancer TMAs (HLug-S120CS01 and HLug-A098Bc01) were obtained from Shanghai Outdo Biotech Co., Ltd for IHC analyses. HPan-S120CS01 included paired adjacent nontumor tissues and 60 cases of lung squamous cell carcinoma tissues. HLug-A098Bc01 included 55 cases of lung adenocarcinoma and 43 normal tissues. The tissue arrays were deparaffinized and rehydrated following previously published procedures (14). Subsequently, we incrementally incubated the tissues with SLC35A2 polyclonal antibodies (1:200, Proteintech) at 4°C overnight. Then slides were washed with cold phosphate-buffered saline (PBS). The sections were flicked, dried, and incubated with biotin-conjugated secondary antibodies for 1h, then revealed by horseradish peroxidase complexes. The sections were visualized using diaminobenzidine. Three pathologists, who knew nothing about the patient clinical features, scored the stained sections. The mean density was calculated as the ratio of integrated optical density (IOD) to the area, and the average mean density of five random areas of the sample was considered the score for that sample. Ethical approval of this study was obtained from the Clinical Research Ethics Committee of Jiangmen Central Hospital (Approval number: 2022–098).

### 2.5 Survival prognosis analysis

Based on the median expression of SLC35A2 in different cancer types, samples were categorized as either SLC35A2-high or SLC35A2-low expression groups. Subsequently, Kaplan–Meier survival analysis was performed to investigate the association between SLC35A2 expression and the disease-specific survival (DSS), disease-free interval (DFI), progression-free interval (PFI), and overall survival (OS) of multiple carcinomas.

### 2.6 Construction of interactive network diagram

The gene set variation analysis was performed on tumor tissues to investigate the modulatory roles of SLC35A2 in oncogenesis. An interaction network of relevant genes was generated by the STRING website (https://cn.string-db.org/). The cut-off value of the interaction score was set at >0.8. The hub genes were obtained using Cytoscape software.

### 2.7 Pan-cancer evaluation of immune cell infiltration, TMB, and MSI status

To estimate tumor immune activity, we calculated immune and stromal scores for each sample using R package “estimation”. The association between SLC35A2 expression and chemokines, immunomodulators, major histocompatibility complexes (MHCs), receptors, and immune checkpoints in human cancers was visualized using the R package “pheatmap”. To ensure accurate results, we downloaded the immune infiltration data of 33 cancers using the TIMER2 web server and evaluated the data using seven algorithms, including CIBERSORT (15), CIBERSORT-ABS (16), EPIC (17), MCP-counter (18), quanTIseq (19), xCell (20), and TIMER (21). We similarly accessed the immune regulation steps *via* TIP (Tracking Tumor Immunophenotype database, http://biocc.hrbmu.edu.cn/TIP/) (22). The TISIDB database (Tumor-Immune System Interaction Database, http://cis.hku.hk/TISIDB/index.php) was applied to evaluate the correlation between tumor-infiltrating lymphocytes (TILs) and the expression level, the normalized copy number, and DNA methylation of SLC35A2. The correlation between SLC35A2 expression and TMB/MSI was evaluated by Spearman’s correlation analysis.

### 2.8 Prediction of response to immune checkpoint blockade therapy

The TIDE algorithm (http://tide.dfci.harvard.edu/) uses a range of markers to evaluate two mechanisms underlying tumor immune evasion: the dysfunction of tumor-infiltrating cytotoxic T lymphocytes (CTLs) and the exclusion of CTLs by immunosuppressive factors. Thus, TIDE is widely used to assess the potential response to ICB therapy. We used four independent immunotherapy cohorts, including GSE35640 (N = 36), GSE61676 (N = 71), GSE78220 (N = 24), and a phase II immunotherapy cohort applied to locally advanced or metastatic uroepithelial cancers (IMvigor210, N = 298), to evaluate the association between SLC35A2 and immunotherapy response (23).

### 2.9 Potential drugs and corresponding targets

Compound sensitivity data was acquired using the CellMiner database (https://discover.nci.nih.gov/cellminer) to analyze the relationship between SLC35A2 expression and antitumor drug sensitivity. We collected two-dimensional (2D) structures using the PubChem database (https://pubchem.ncbi.nlm.nih.gov) and then transformed them into 3D structures using ChemBio3D Ultra 13.0. The 3D protein structure of the core target protein was extracted using the UniProt database (https://www.uniprot.org/). Molecular docking was performed using Autodock-vina1.1.2 software. Discovery Studio 2019 was conducted to visualize the docking results.

## 3 Results

### 3.1 Genetic alteration of SLC35A2 in human cancers

**Figure 1:**
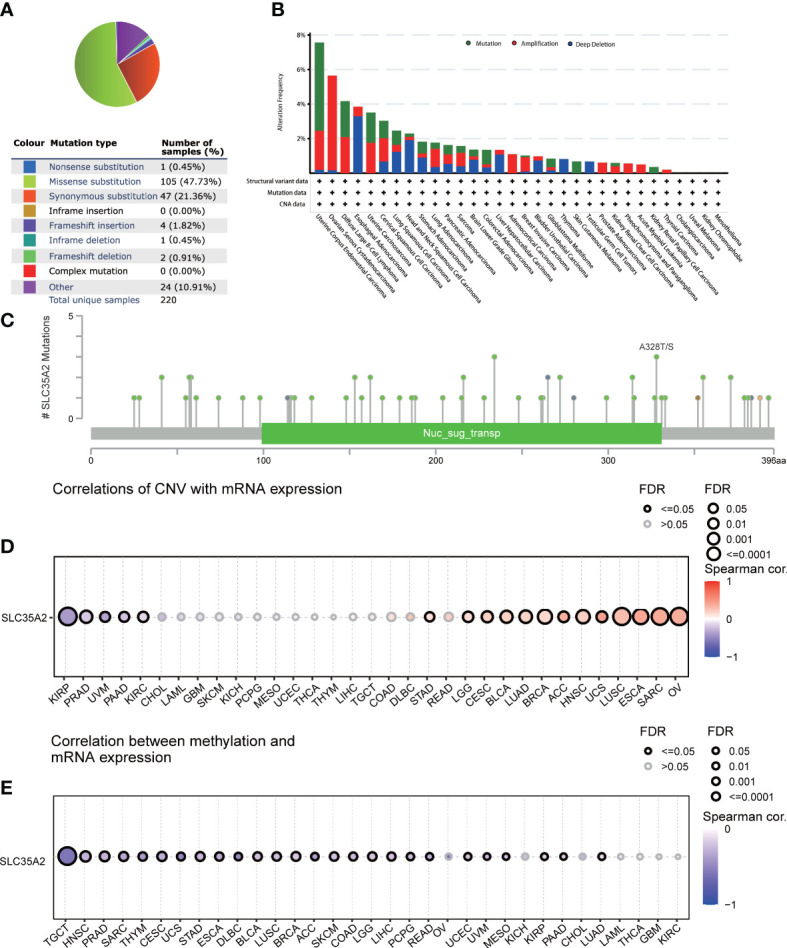
Mutational landscape of solute carrier family 35 member A2 (SLC35A2) in multiple cancers **(A)** Distribution of different types of mutations in SLC35A2. **(B)** Frequency of different SLC35A2 genetic alterations in multiple cancers. **(C)** Mutation types and sites of alteration frequency in SLC35A2. **(D)** Spearman’s correlation analysis showed the association between copy number variation and SLC35A2 expression. **(E)** Spearman’s correlation analysis showed the association between methylation and SLC35A2 expression. FDR, false discovery rate; calculated by adjusting the P value in Spearman’s analysis with the BH method. Significant were determined by FDR ≤ 0.05.

A series of mutations in key regulatory genes give rise to cancer initiation and development. To study the genetic mutation of SLC35A2, we investigated the distribution of different somatic mutations that occurred in SLC35A2 using the COSMIC database. Among 670 cancer samples tested, the most common mutation type was missense substitutions at 47.73% (**Figure 1A**). We subsequently analyzed SLC35A2 genetic alteration in multiple cancers using the cBioPortal tool. It came out that uterine corpus endometrial carcinoma (UCEC) is the cohort with the highest SCL35A2 alterations frequency, among which mostly were mutations, followed by patients with ovarian serous cystadenocarcinoma (OV) (**Figure 1B**). We also noted that the alanine to threonine or serine mutations at 328 positions of the SLC35A2 protein had the highest frequency among the mutations (**Figure 1C**). Next, we used GSCA to explore whether CNV and DNA methylation in SLC35A2 affected its expression in 33 cancers. The findings revealed a positive association between CNV and SLC35A2 expression in 13 cancers, including BLCA, LUAD, and BRCA (considered significant when FDR ≤ 0.05). CNV and SLC35A2 expression were negatively correlated in five cancers, such as pancreatic adenocarcinoma (PAAD) (considered significant when FDR ≤ 0.05), suggesting that SLC35A2 expression was primarily regulated by other factors such as the activation of transcription in these cancers (**Figure 1D**). Spearman’s correlation analysis was conducted to explore the effects of DNA methylation on SLC35A2 expression. A negative relationship between SLC35A2 expression and DNA methylation was found in 26 cancers (**Figure 1E**). Furthermore, the SLC35A2 promoter region was hypomethylated in testicular germ cell tumors (TGCT), BLCA, BRCA, esophageal carcinoma (ESCA), LIHC, prostate adenocarcinoma (PRAD), and stomach adenocarcinoma (STAD) (**Supplementary Figure 1**). Using the DNMIVD tool, we assessed the association between SLC35A2 methylation and cancer patient prognosis. A significant relationship was revealed between the high level of SLC35A2 methylation and worse OS in head and neck squamous cell carcinoma (HNSC) and kidney renal papillary cell carcinoma (KIRP) (**Supplementary Figure 2**). We then used TIMER2 to compare the expressions of substrate genes based on SLC35A2 mutations. These results indicated that SLC35A2 mutations were associated with the expression of substrate genes in SKCM, COAD, and LUSC; we found an increased expression of UGT1A4 in COAD samples, an increased expression of UGT1A7 in SKCM samples, and a decreased expression of UGT1A3 in LUSC samples with mutated SLC35A2 (**Supplementary Figure 3**). Therefore, DNA copy number amplification and methylation are two factors that may contribute to changes in SLC35A2 expression in cancers.

### 3.2 Pan-cancer expression landscape of SLC35A2

**Figure 2:**
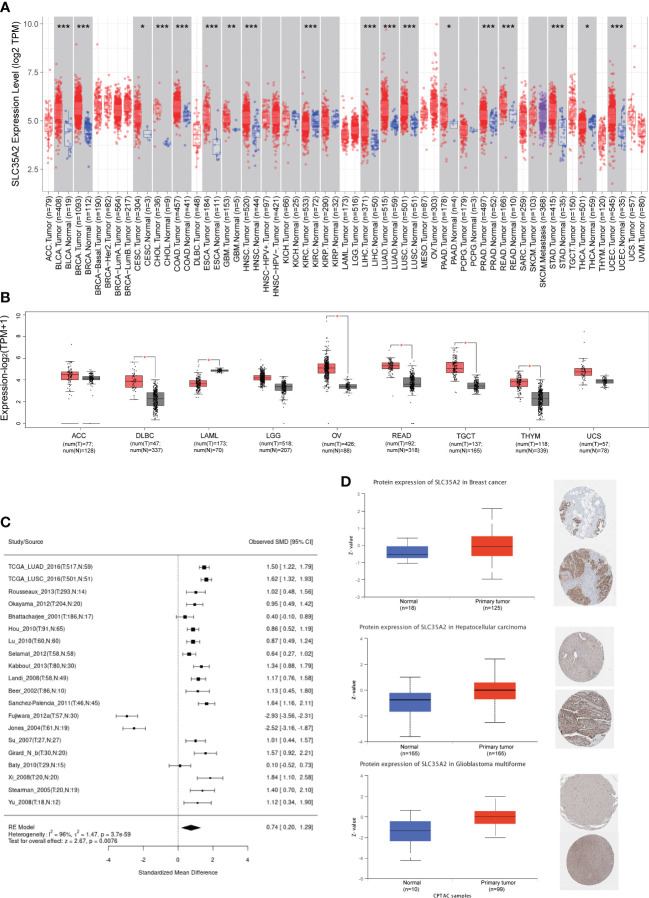
Expression of solute carrier family 35 member A2 (SLC35A2) mRNA and their protein levels in tumors and healthy tissues. **(A)** SLC35A2 mRNA expression in multiple cancers from The Cancer Genome Atlas (TCGA)database. **(B)** SLC35A2 mRNA expression in tumor and normal tissues from TCGA and Genotype-Tissue Expression (GTEx) databases. Differences between means were determined by Student’s t-test, *P < 0.05; **P < 0.01; ***P < 0.001 **(C)** A meta-analysis for comparing the difference in SLC35A2 expression between normal and tumor tissues. **(D)** SLC35A2 protein levels between BRCA, LIHC, GBM, and their corresponding normal tissues were explored from CPTAC and HPA databases.

**Figure 3:**
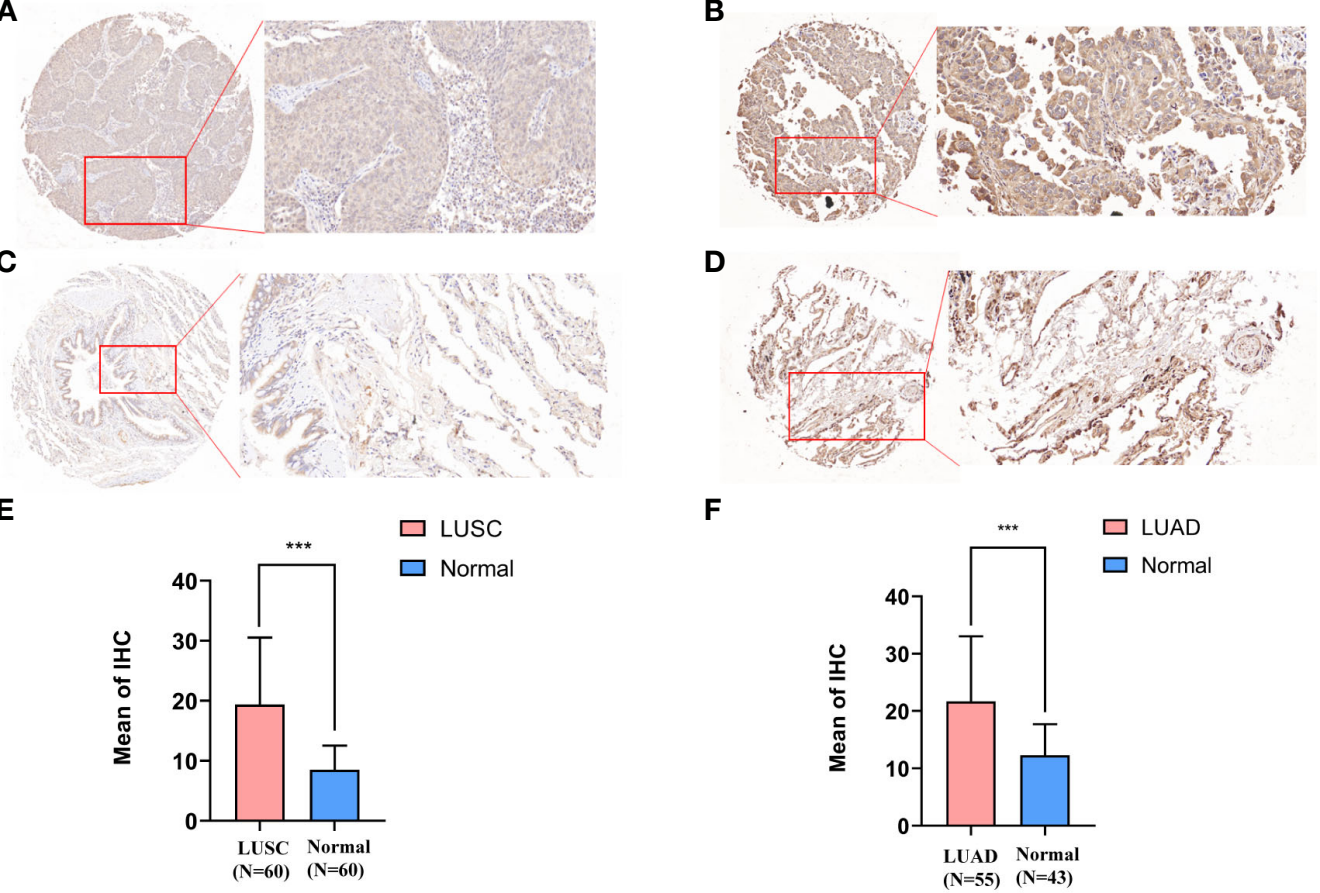
Immunohistochemical staining results and statistics of solute carrier family 35 member A2 (SLC35A2) protein in lung adenocarcinoma (LUAD) and squamous cell carcinoma. **(A, C)** The 20× immunohistochemical images of lung squamous cell carcinoma (LUSC). **(B, D)** The 20× immunohistochemical images of LUAD. **(E)** The statistical results of LUSC and normal lung tissues. **(F)** The statistical results of LUAD and normal lung tissues (Student’s *t*-test; ***P < 0.001).

The expression of SLC35A2 was explored in various healthy tissues and cell lines by the HPA database. The highest expression of SLC35A2 was observed in the fallopian tube and the lowest in the retina (**Supplementary Figure 4A**). Respiratory ciliated cells had the highest SLC35A2 levels compared to other cell types (**Supplementary Figure 4B**). Subsequently, we investigated the changes in SLC35A2 expression in multiple cancers. It suggested that SLC35A2 was highly expressed in 16 cancers [LUAD, LIHC, cervical squamous cell carcinoma and endocervical adenocarcinoma (CESC), PRAD, GBM, HNSC, BLCA, LUSC, cholangiocarcinoma (CHOL), STAD, UCEC, rectum adenocarcinoma (READ), PAAD, BRCA, COAD, and ESCA] than in adjacent healthy tissues (P < 0.05, **Figure 2A**). We matched the GTEx dataset as controls for cancer types without healthy samples or those with less than two healthy samples. SLC35A2 was expressed higher in lymphoid neoplasm diffuse large B-cell lymphoma (DLBC), OV, TGCT, and thymoma (THYM) than in the corresponding healthy tissues. Acute myeloid leukemia (LAML) expressed lower SLC35A2 than in healthy tissues (P < 0.05, **Figure 2B**). Furthermore, increased SLC35A2 expression was noted in uterine carcinosarcoma (UCS), brain lower grade glioma (LGG), adrenocortical carcinoma (ACC), and pheochromocytoma and paraganglioma (PCPG); however, the differences were not statistically significant. Furthermore, we conducted a “tumor versus normal” meta-analysis of different lung cancer cohorts using the LUNG CANCER EXPLORER. SLC35A2 expression was found to be upregulated (observed SMD > 0) in the majority of the datasets (**Figure 2C**). SLC35A2 methylation was also inversely associated with mRNA expression in BRCA, ESCA, LIHC, PRAD, BLCA, TGCT, and STAD. Using the UALCAN database, we evaluated the post-translational level of SLC35A2. Compared to healthy tissues, higher SLC35A2 protein expression levels were demonstrated in GBM, HNSC, and BRCA. We further analyzed IHC images from the HPA database. Normal breast, liver, and cortical tissues were negative for SLC35A2 IHC staining, whereas tumor tissues were intensely stained (**Figure 2D**). We also investigated the SLC35A2 protein levels in cohorts of patients with LUSC and LUAD using IHC (HLug-A098Bc01 and HLug-S1230CS01). The SLC35A2 levels were considerably higher in LUAD (P < 0.001) and LUSC (P < 0.001) than in corresponding healthy tissues (**Figures 3A–F**). In summary, SLC35A2 expression was upregulated in various cancers, suggesting that high levels of SLC35A2 may be correlated with tumor progression.

### 3.3 Pan-cancer correlations of SLC35A2 level with clinicopathological features and prognosis

**Figure 4:**
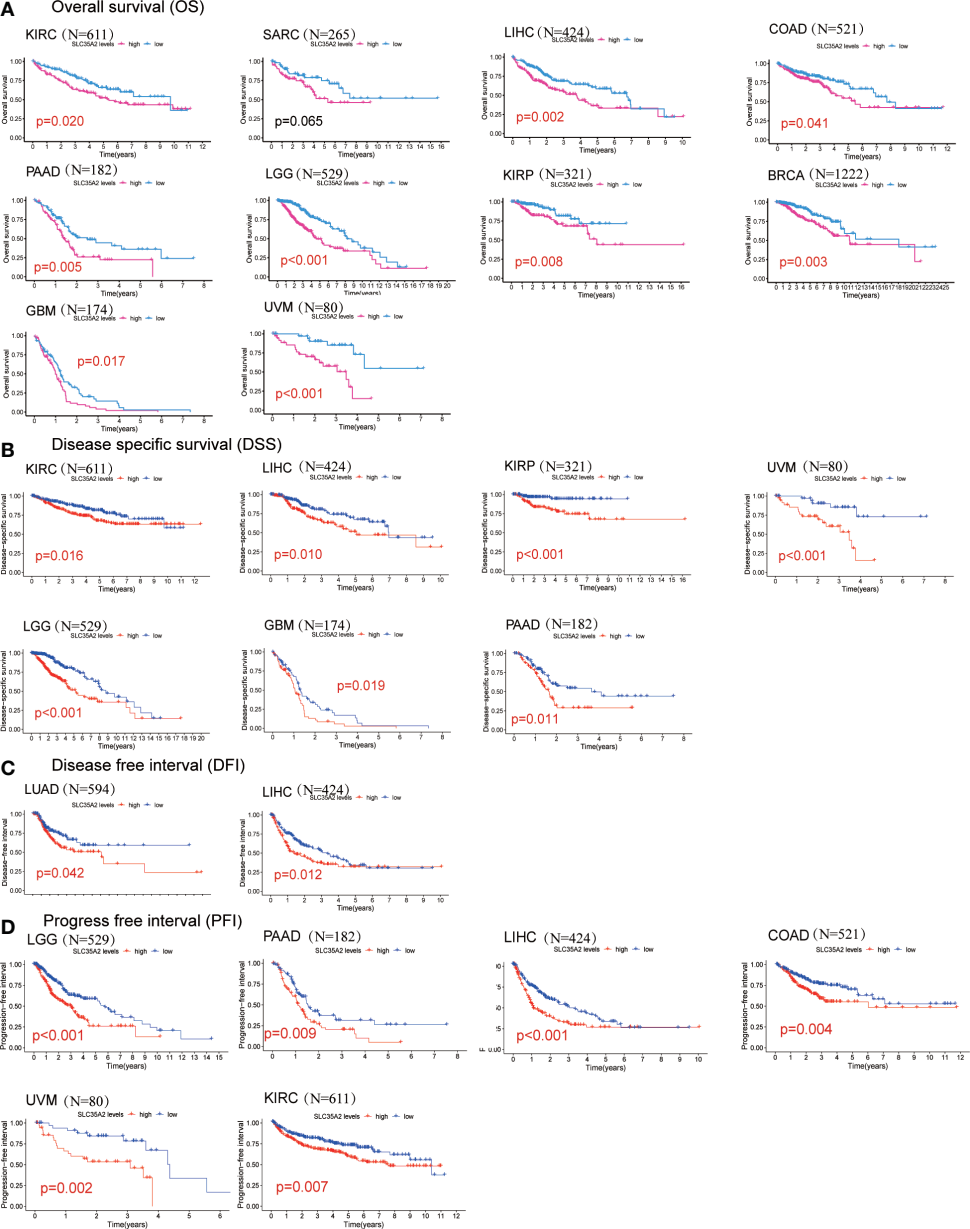
Kaplan–Meier survival curves comparing the high and low expression of solute carrier family 35 member A2 (SLC35A2) in various cancer types. **(A)** Overall survival of 10 cancer types. **(B)** Disease-specific survival of 7 cancer types. **(C)** Disease-free interval of 2 cancer types. **(D)** Progression-free interval of 6 cancer types. A log-rank test was used to analyze the significance of differences between groups.

**Figure 5:**
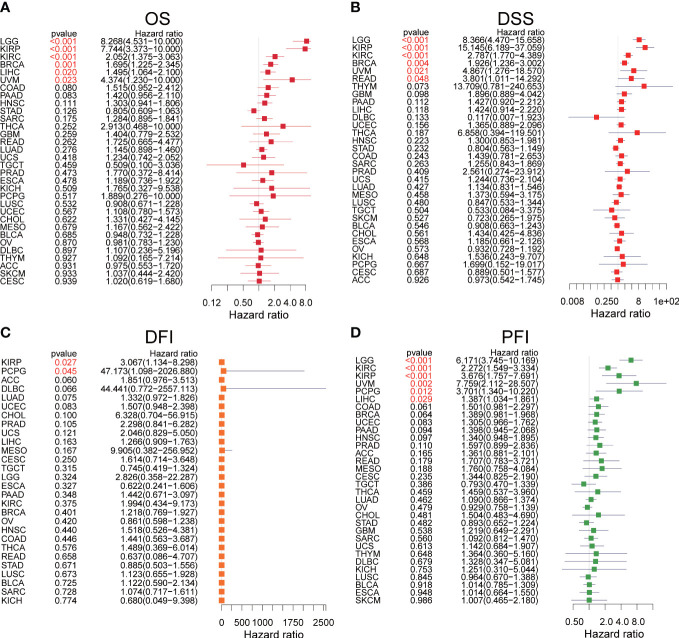
The forest plots of univariate Cox regression analyses. **(A)** The results of solute carrier family 35 member A2 (SLC35A2) for overall survival in pan-cancer. **(B)** The results of SLC35A2 for disease-specific survival in pan-cancer. **(C)** The results of SLC35A2 for the disease-free interval in pan-cancer. **(D)** The results of SLC35A2 for the progression-free interval in pan-cancer. A log-rank test was used to analyze the significance of differences between groups.

We explored the prognostic significance of SLC35A2 using TCGA datasets. The results revealed that increased expression of SLC35A2 led to the worse prognosis of nine tumors, including kidney renal clear cell carcinoma (KIRC), LIHC, COAD, PAAD, LGG, KIRP, BRCA, uveal melanoma (UVM), and GBM (**Figure 4A**). The Kaplan–Meier survival analysis revealed SLC35A2 as a protective factor in TGCT. Instead, SLC35A2 was a significant risk factor for DSS in seven tumors, DFI in two tumors, and PFI in six tumors (**Figures 4B–D**). To further demonstrate the prognostic predictive value of SLC35A2, Kaplan–Meier survival analysis was applied to explore the correlation between SLC35A2 and prognosis in 4167 patients from 19 independent datasets. The results revealed that patients with overexpression of SLC35A2 had a worse prognosis, including patients with LUAD, LGG, UCS, LAML, sarcoma (SARC), SKCM, READ, LIHC, LUSC, KIRP, GBM, HNSC, ESCA, OV, DLBC, BLCA, BRCA (HER2-positive subtype), BRCA (TNBC subtype), and mesothelioma (MESO) (**Supplementary Figure 5**). We performed Cox regression analyses to confirm and observe that SLC35A2 levels were highly associated with OS in six tumors, DSS in six tumors, DFI in two tumors, and PFI in six tumors (**Figures 5A–D**). We also assessed the association between tumor stages and SLC35A2 expression. It was revealed that the high expression of SLC35A2 and advanced clinical stages were highly relevant in 18 cancers, including STAD, PAAD, LUAD, KIRC, ESCA, BRCA, thyroid cancer (THCA), TGCT, READ, LUSC, KIRP, HNSC, ACC, SKCM, LIHC, kidney chromophobe (KICH), COAD, and BLCA (**Supplementary Figure 6**).

### 3.4 Gene set and functional enrichment analysis

We evaluated the correlation between SLC35A2 and cancer hallmark pathway scores. SLC35A2 was predominantly related to the mammalian target of rapamycin complex 1 across 33 cancer types (**Supplementary Figure 7A**). Next, we constructed a protein-protein interactional (PPI) network by mining the STRING database (**Supplementary Figure 7B**). Hub genes, including SDC1, SDC2, SDC3, GPC1, DCN, and VCAN, were significantly associated with SLC35A2 expression.

### 3.5 Pan-cancer tumor immune microenvironment

TILs are essential components of the tumor microenvironment (TME) and contribute to tumorigenesis and progression of cancers. We first calculated the stromal and immune scores using the “ESTIMATE” R package and conducted a correlation analysis to understand the association between SLC35A2 and TME. An inverse correlation is observed between SLC35A2 expression and immune score in seven cancer types, including THYM, HNSC, PAAD, PRAD, STAD, LUAD, and COAD (P < 0.05, **Supplementary Figure 8**). The relevance of chemokines, chemokine receptors, immunomodulators, checkpoints, MHCs, and SLC35A2 expression was evaluated using Spearman’s correlation analysis in 33 tumors. The MHCs and other immunomodulator genes were inversely expressed with SLC35A2 in PAAD and STAD (**Supplementary Figure 9A**); this was consistent with the results of the ESTIMATE immune score. SLC35A2 was positively correlated with CD276 and PVR cell adhesion molecule in PAAD and STAD (**Supplementary Figure 9B**). Therefore, in multiple cancers, higher expression of SLC35A2 correlated with low TILs, and this may be related to levels of MHCs and immune checkpoint genes in PAAD and STAD.

To prevent calculative errors originating from applying a single algorithm and distinction of TILs marker gene sets, we used seven algorithms (xCell, TIMER, quanTIseq, EPIC, CIBERSORT-ABS, MCP-counter, and CIBERSORT) to assess the correlation between SLC35A2 expression and levels of immune infiltration. Seven algorithms revealed levels of CD8+ T cell infiltration, five algorithms revealed levels of NK cell infiltration, and three algorithms revealed that SLC35A2 expression in multiple cancers was negatively correlated with B and CD4+ T cells (**Supplementary Figure 10**). A negative association was also revealed between SLC35A2 expression and CD8+ T cell infiltration levels(calculated using the TIMER algorithm) in STAD, THYM, OV, UCEC, SKCM, BRCA, KIRC, LUSC, COAD, READ, and LUAD. Furthermore, SLC35A2 expression and NK cell infiltration levels were negatively correlated as calculated using the MCP-counter algorithm in PRAD, THCA, STAD, COAD, LUSC, READ, LUAD, SKCM, BRCA, KIRC, KIRP, and SARC. Increased SLC35A2 expression may be correlated with immunodepression in the tumor microenvironment.

**Figure 6:**
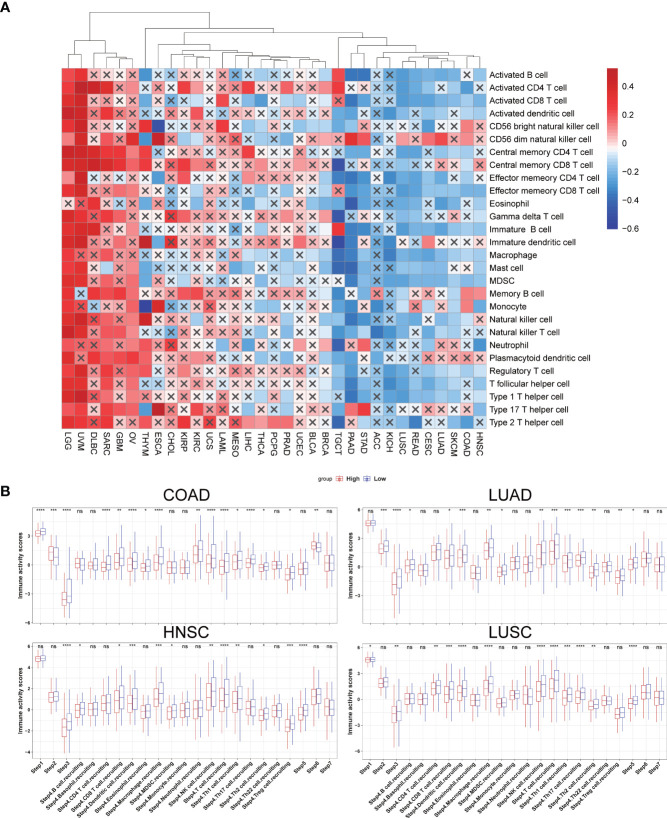
The effect of solute carrier family 35 member A2 (SLC35A2) on immunological status in cancers. **(A)** Correlation between SLC35A2 and immune cells using the single-sample GSEA (ssGSEA)algorithm. **(B)** Differences in the steps of the cancer immune cycle between groups with high and low SLC35A2 expression. Differences between means were determined by Student’s *t*-test. ns, no significant difference; *P < 0.05; **P < 0.01; ***P < 0.001; ****P < 0.0001.

The process flows of the immunity cycle could be summarized as a sequence of events: releasing cancer cell antigens, presenting cancer antigens, priming and activation, trafficking and infiltrating immune cells to tumors, recognizing tumor antigens, and killing cancer cells. Therefore, we performed the single-sample gene set enrichment analysis (ssGSEA) to investigate the involvement of SLC35A2 in the immune activation process. The expressions of SLC35A2 in PAAD, READ, SKCM, STAD, CESC, LUAD, COAD, LUSC, and HNSC were negatively related to the level of multiple immune cell infiltration (**Figure 6A**). When the SLC35A2 expression was low in LUSC, COAD, HNSC, and LUAD, the steps of immune response were activated (**Figure 6B**).

**Figure 7:**
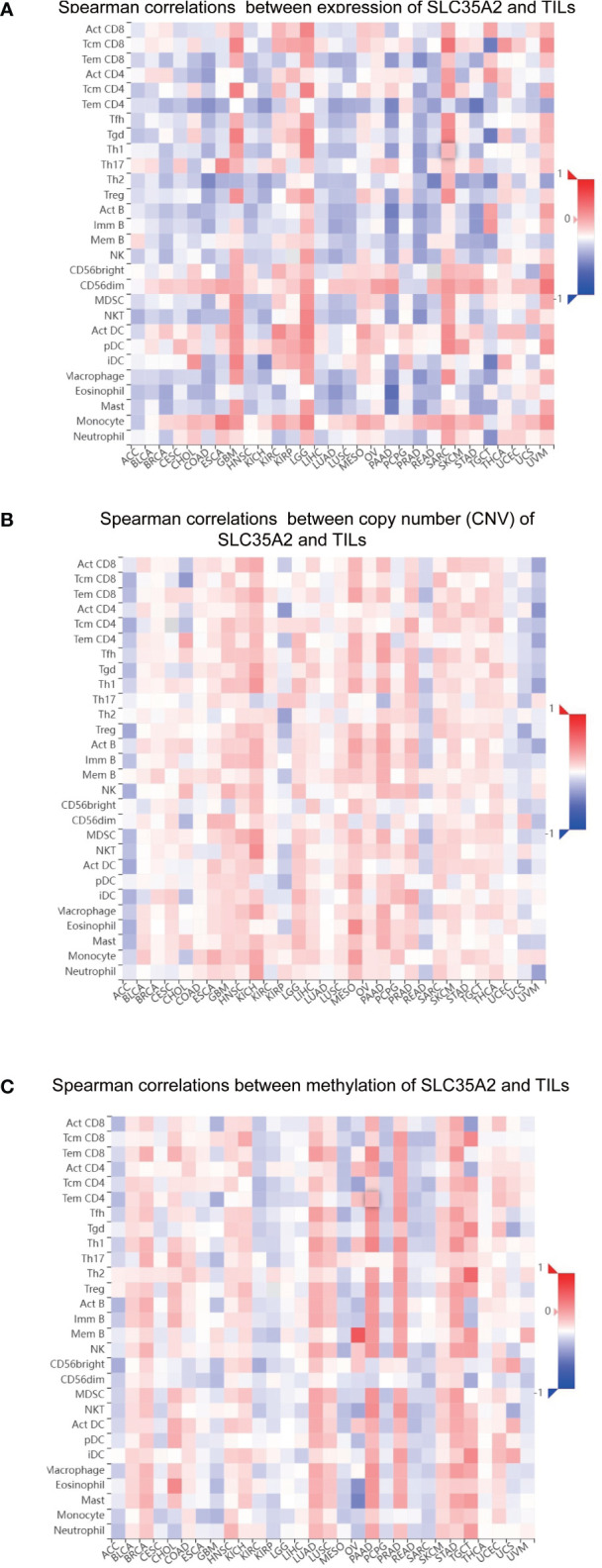
Spearman correlation heatmap showing the relationship between the abundance of tumor-infiltrating lymphocytes (TILs) and **(A)** solute carrier family 35 member A2 (SLC35A2) expression, **(B)** copy number variation (CNV), and **(C)** methylation in the TISIDB database.

Correlations were sought between TIL abundance and SLC35A2 expression, methylation, and copy number using the TISIDB database. TIL abundance was inversely related to the expression of SLC35A2 in TGCT, PRAD, READ, LUAD, PAAD, ACC, BRCA, KICH, COAD, STAD, LUSC, and HNSC (**Figure 7A**). Furthermore, the CNV of SLC35A2 and TILs in ACC, KIRP, READ, UCS, and UVM were negatively correlated (**Figure 7B**). SLC35A2 methylation was positively associated with TIL abundance in PRAD, BRCA, BLCA, PAAD, STAD, and LUAD (**Figure 7C**). Thus, SLC35A2 is essential for tumor immune regulation in the abovementioned tumors.

### 3.6 Pan-cancer correlations of SLC35A2 expression with MSI with TMB

**Figure 8:**
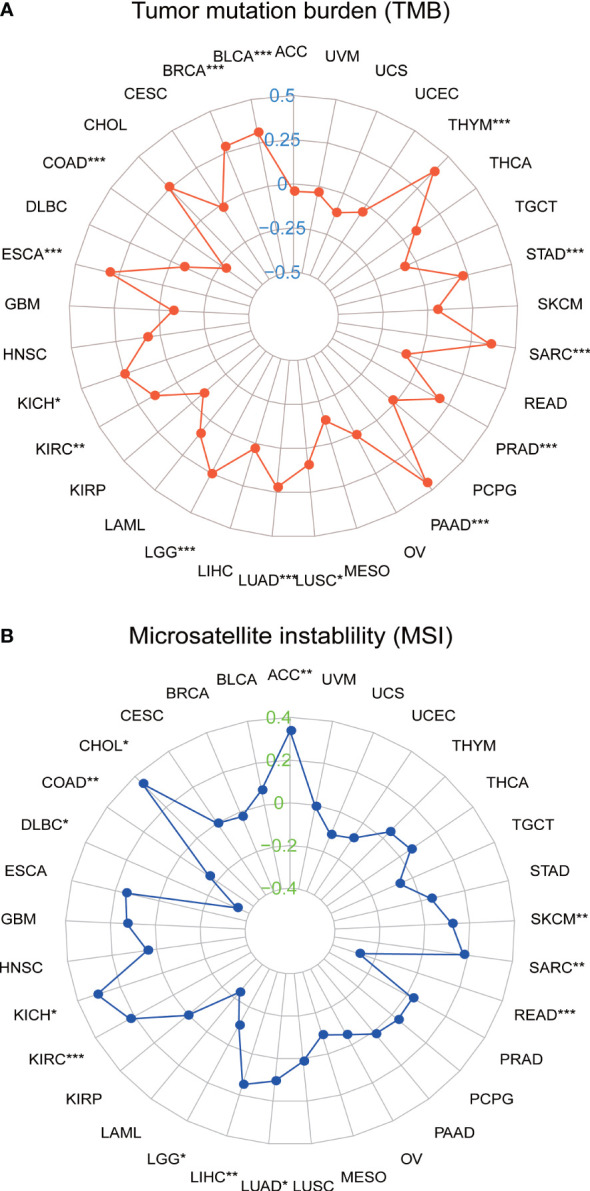
Correlation between solute carrier family 35 member A2 (SLC35A2) expression and **(A)** tumor mutational burden (TMB) and **(B)** microsatellite instability (MSI). Spearman’s correlation analysis was used to analyze the association *P < 0.05; **P < 0.01; ***P < 0.001.

We further investigated the relationship between SLC35A2 expression and TMB/MSI in all tumors. SLC35A2 expression was negatively correlated with TMB in COAD (P = 1.1e−08), whereas a positive correlation was observed in KIRC (P = 0.007), BLCA (P = 1.5e−10), BRCA (P = 1.6e−19), ESCA (P = 7.8e−05), LGG (P = 8.8e−09), LUSC (P = 0.036), PAAD (P = 7.6e−09), KICH (P = 0.044), PRAD (P = 5.6e−05), SARC (P = 1.9e−08), THYM (P = 2.3e−05), LUAD (P = 4.4e−07), and STAD (P = 3.3e−05) (**Figure 8A**). Moreover, there was an inverse correlation observed between the expression of SLC35A2 and MSI in LGG (P = 0.021), DLBC (P = 0.018), READ (P = 0.0006), and COAD (P = 0.001) (**Figure 8B**), whereas there was a positive correlation in SARC (P = 0.004), CHOL (P = 0.036), SKCM (P = 0.009), KICH (P = 0.012), LIHC (P = 0.004), LUAD (P = 0.017), ACC (P = 0.002), and KIRC (P =3.9e−05). These results revealed that SLC35A2 may be an indicator of cancer immunogenicity in the abovementioned cancer types.

### 3.7 Analysis of immunotherapy response

**Figure 9:**
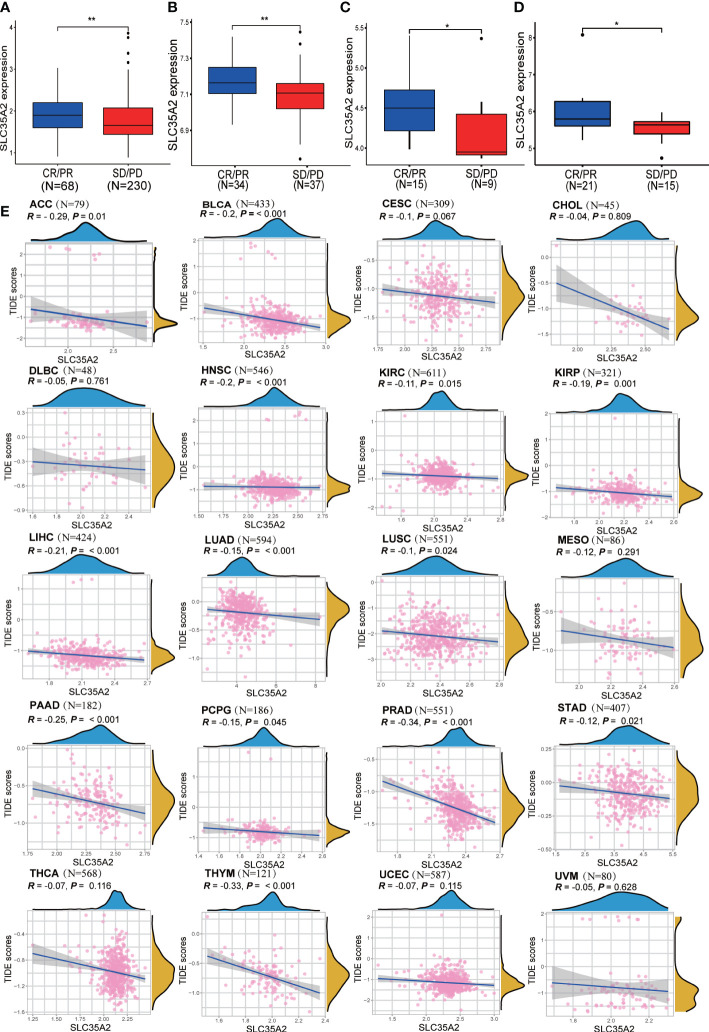
Exploring the relationship between solute carrier family 35 member A2 (SLC35A2) expression and immunotherapy response. **(A)** A box plot was comparing SLC35A2 expression with immune checkpoint blockade (ICB) response in the IMvigor210 cohort. **(A)** A box plot was comparing SLC35A2 expression with ICB response in the IMvigor210 cohort. **(B)** A box plot comparing SLC35A2 expression with ICB response in the GSE61676. **(C)** A box plot comparing SLC35A2 expression with ICB response in the GSE78220. **(D)** A box plot comparing SLC35A2 expression with ICB response in the GSE35640. **(E)** The Spearman’s correlation analysis showed an association between SLC35A2 expression and the TIDE scores. Differences between means were determined by Student’s *t*-test. *P < 0.05; **P < 0.01.

We verified the relationship between SLC35A2 expression and ICB response with four independent immunotherapy cohorts. SLC35A2 expression was higher in patients exhibiting complete and partial ICB responses than in those exhibiting stable and progressive disease (P < 0.01, **Figures 9A–D**). Moreover, the TIDE score is an essential index for predicting immunotherapy response. A negative correlation was observed between the expression of SLC35A2 and the TIDE score in 20 tumors, including BLCA, LUAD, and LUSC, suggesting the expression of SLC35A2 is correlated with ICB response and may serve as a marker for ICB treatment (**Figure 9E**). Taken together, patients with high SLC35A2 expression may benefit from immunotherapy.

### 3.8 Identification of SLC35A2-related drugs

**Figure 10:**
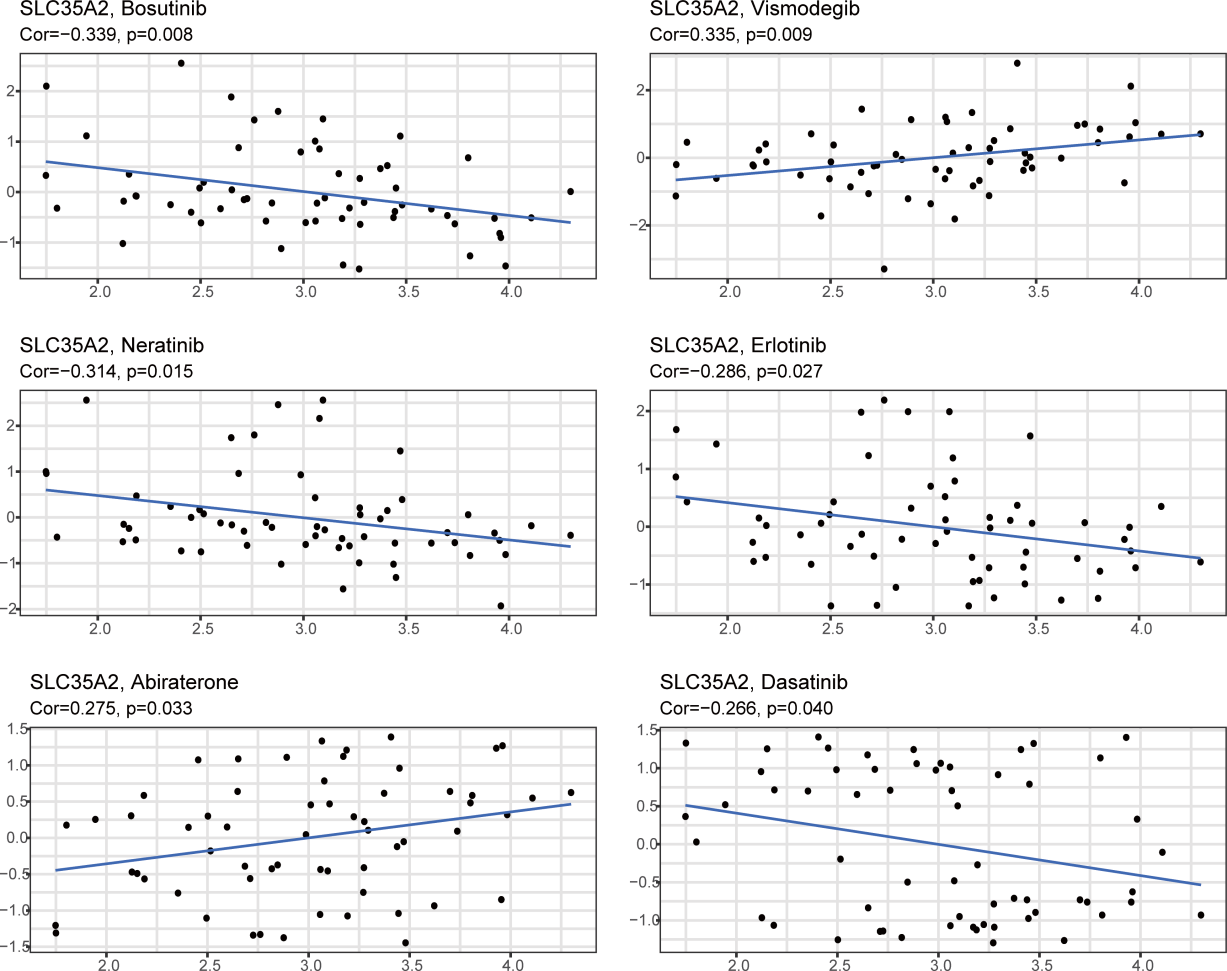
The plot showed the correlation between the expression of solute carrier family 35 member A2 (SLC35A2) and drug sensitivities.

**Figure 11:**
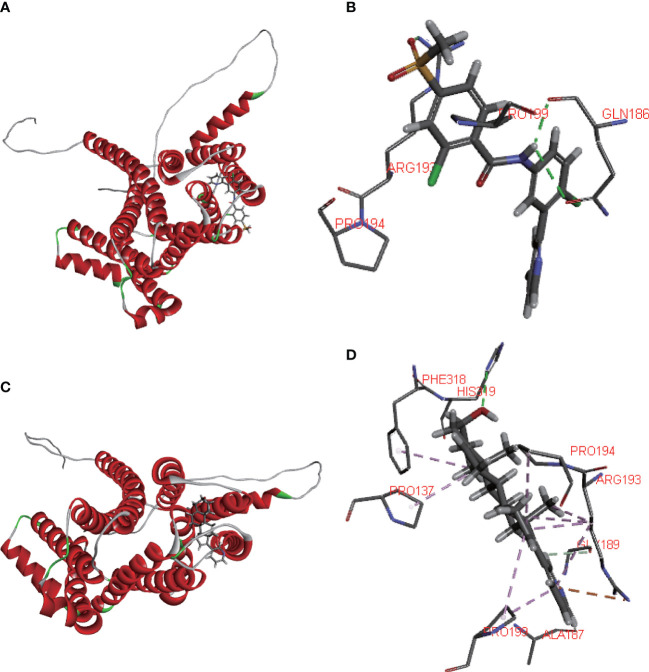
Molecular docking. **(A, B)** 2D and 3D diagrams showing the molecular docking of solute carrier family 35 member A2 (SLC35A2) to vismodegib. **(C, D)** 2D and 3D diagrams showing the molecular docking of SLC35A2 to abiraterone.

Using the CELLMINER drug response data, our findings indicated that the expression of SLC35A2 was positively correlated with the susceptibility of vismodegib and abiraterone (**Figure 10**, hazard ratio > 1, P < 0.05). Conversely, SLC35A2 expression was negatively associated with the sensitivity of bosutinib, neratinib, erlotinib, and dasatinib. To further explore the binding of vismodegib and abiraterone with SLC35A2, we conducted molecular docking studies. The free binding energy of SLC35A2 with vismodegib was −0.191 kcal/mol. The binding affinity was attributed to the Van der Waals interactions with the RPO194 residue and hydrophobic interactions with the ARG193 and GLN186 residues (**Figures 11A, B**). The free binding energy of SLC35A2 with abiraterone was −9.771 kcal/mol. The binding affinity was attributed to the Van der Waals interactions with the GLY189 residue, hydrophobic interactions with the HIS319 residue, and pi-alkyl interactions with the ARG193 residue (**Figures 11C, D**). We also studied the molecular docking of abiraterone with its target protein CYP17 (**Supplementary Figure 11**), and the results indicated that the validated interaction of CYP17/abiraterone has a higher free binding energy (−7.2 kcal/mol) than SLC35A2/abiraterone (−9.771 kcal/mol). Thus, an intermolecular binding may exist between SLC35A2 and abiraterone. Therefore, unveiling the expression and regulation of SLC35A2 in cancers has a guiding significance in pharmacotherapy options in clinical practice.

## 4 Discussion

Genetic and epigenetic alterations contribute to cancer oncogenesis and progression (24). Glycosylation is the most frequently observed post-translational modification in -membrane-bound proteins, playing an essential role in cell proliferation, translation, and adhesion (25). We have previously developed a novel signature for breast cancer (26). However, whether SLC35A2 plays an essential role in different cancers *via* a common underlying molecular mechanism remains unclear. Therefore, we comprehensively analyzed the expression of SLC35A2 across multiple cancers in multiple datasets.

Identifying gene mutation is critical for cancer-related diagnosis and therapeutic decision-making. The findings revealed that missense substitution was the most common type of mutation, and patients with UCEC exhibited the highest frequency of SCL35A2 alterations. The CNV of SLC35A2 showed a positive association with 13 cancer types. Conversely, the CNV of SLC35A2 showed a negative association with five cancer types. Altogether, these findings suggest that genetic alterations serve an essential role in regulating SLC35A2 expression and patient prognosis. However, the underlying regulatory mechanisms require a more comprehensive and in-depth investigation.

To determine the gene expression landscape of SLC35A2 in pan-cancer, we integrated different independent datasets from TCGA and GTEx databases. Furthermore, we found the overexpression of SLC35A2 in multiple tumor tissues, including LUAD, GBM, PRAD, STAD, COAD, ESCA, BRCA, UCEC, HNSC, LUSC, CHOL, PAAD, LIHC, READ, CESC, and BLCA, DLBC, OV, TGCT, and THYM, but not in the corresponding healthy tissues. It suggested that SLC35A2 as a significant biological maker involved in various cancer. The protein levels of SLC35A2 were higher in LUAD, LUSC, GBM, HNSC, and BRCA than those in corresponding normal tissues. Previous studies have indicated that copy number amplification correlates with upregulated gene expression. However, this result may be driven by a sophisticated regulatory mechanism between gene expression and CNV (27).

DNA methylation is one of the most common epigenetic modifications involved in tumorigenesis (28). Based on the UALCAN tool, we identified the promoter hypomethylated status in SLC35A2 in 7 cancer types compared to healthy tissues. This is consistent with the high expression of SLC35A2 in various cancers. Furthermore, we studied the link between SLC35A2 methylation levels and OS in patients with cancer. We found that the methylation level of SLC35A2 affected the prognosis of three types of malignancies.

To comprehensively determine the predictive significance of SLC35A2 expression in multiple cancers, we conducted a Kaplan–Meier survival analysis to evaluate the correlation between SLC35A2 expression and the prognosis of patients in the TCGA, METABRIC, CGGA, and GEO databases. In our study, patients with a high expression of SLC35A2 had worse prognoses, including patients with BRCA (HER2-positive subtype), BRCA (TNBC subtype), COAD, GBM, LIHC, and PAAD. These findings suggested that SLC35A2 could serve as a multifaceted biomarker for prognosis in pan-cancer and the high expression of SLC35A2 might be associated with unfavorable clinical outcomes in various tumors.

TILs should be explored in-depth as essential biomarkers for predicting cancer treatment efficacy. This study showed that cancer immunity is closely associated with stromal and immune scores. We found that SLC35A2 expression negatively correlates with the immune score of seven cancers and the stromal score of nine cancer types. Furthermore, the interplay between tumor cells and immunity is a comprehensive regulatory network consisting of the chemokine system and immune regulators (29). Increased immune checkpoint genes, such as PD-1 or PD-L1, were significantly associated with a poor prognosis and responsiveness to immunotherapy (30). Exploring the correlation between the expression of immune checkpoint genes and SLC35A2 expression can help understand the prognosis and determine the response to immunotherapy in patients with these cancers. These findings indicated that these genes were positively associated with SLC35A2 expression in many cancers, especially UVM. Notably, SLC35A2 is speculated to be involved in the immune regulation of TME, and patients with UVM having high SLC35A2 expression may respond relatively well to immunotherapy. We then used seven algorithms (xCell, TIMER, quanTIseq, EPIC, CIBERSORT-ABS, MCP-counter, and CIBERSORT) to determine the correlation between SLC35A2 expression and immune infiltration levels of TILs. The activated CD8+ TILs can affect the antitumor immune response and prevent tumor progression at an early stage by directly attacking tumor cells (31). A clinical trial found an increase in the complete pathological response associated with highly infiltrated CD8+ TILs (32). NK cells increase the abilities of T cells and other immune cells by producing various cytokines and chemokines (33). We identified that SLC35A2 expression is negatively associated with the levels of CD8+ T cell infiltration, NK cell infiltration, B cells, and CD4+ T cells in various cancers, indicating that higher SLC35A2 expression may be correlated to fewer lymphocyte cells infiltration and lead to poor prognosis. Considering the complicated process of tumor immune response, we assessed the steps of immune activation by the TIP algorithm. We found downregulation of SLC35A2 had higher activation scores, including LUSC, HNSC, COAD, and LUAD. This indicated the good prognosis of patients with lower SLC35A2 expression, possibly due to increased lymphocytes and immune activation within the microenvironment.

TMB is a good predictive indicator of response to ICB therapy. Many previous studies have shown that TMB could predict the responsive and survival benefit of checkpoint blockade (34, 35). Similarly, MSI, an indicator of genetic instability, is becoming more commonly utilized to identify patients who might benefit from immunotherapy, targeted therapies, and advanced systemic therapeutic approaches (36). There is a tendency to benefit from immunotherapy for long-term survival in high TMB or MSI patients (37, 38). To date, no study has been performed on TMB and MSI for SLC35A2. We explored the correlation between SLC35A2 expression and TMB/MSI. Our findings confirmed a positive association in SLC35A2 expression with TMB in 14 types of cancer, and MSI in 12 types of cancer. In COAD, SLC35A2 expression is negatively correlated with both MSI and TMB, whereas it is positively correlated in KIRC, LUAD, and SARC. This suggests that SLC35A2 may potentially affect the immunotherapy response of COAD, KIRC, LUAD, and SARC. To summarize, these findings suggested that SLC35A2 likely plays a critical role in the recruitment and regulation of TILs in cancers. In other words, SLC35A2 is involved in tumor immune evasion and cancer progression by downregulating the number of activated tumor-infiltrating lymphocytes, such as CD8+ T cells, CD4+ T cells, NK cells, and B cells, and ultimately affecting patient survival.

The TIDE score is widely accepted and recommended for assessing immune response. It is related to T-cell rejection in low-CTL tumors and T-cell dysfunction in high-CTL tumors, thus representing two distinct immune evasion mechanisms. Patients with a higher TIDE score are at a greater risk of experiencing antitumor immune evasion and exhibit a lower response rate to ICB therapy (39). Several previous studies have shown that the TIDE score is a more accurate predictor for patients undergoing ICB therapy compared with PD-L1 expression and TMB (39–42). In this study, we found that SLC35A2 expression is negatively associated with TIDE score in 20 tumors, suggesting that individuals with high levels of SLC35A2 expression had a reduced incidence of immune evasion. Furthermore, we used four independent immune therapy cohorts with a total of 429 patients to determine the correlation between SLC35A2 and ICB response. Our findings revealed that SLC35A2 expression was lower among patients exhibiting stable and progressive disease, while it was higher among patients exhibiting complete and partial response. These findings indicated that SLC35A2 expression is related to ICB response and has the potential as a promising marker for ICB therapy. Subsequently, we developed a molecular docking model showing vismodegib and abiraterone binding modes with SLC35A2. Many previous studies have shown that abiraterone acetate, a selective and irreversible inhibitor of the CYP17 enzyme, can block androgen synthesis and potentially prolong the survival of patients with metastatic castration-resistant prostate cancer. The free binding energy of CYP17 with abiraterone is higher than that of SLC35A2 with abiraterone, indicating that SLC35A2 binds more easily to abiraterone and offers high confidence (43, 44).

Despite our pan-cancer analysis of SLC35A2 using multi-dimensions, our study still has some limitations that should be addressed. The microarray and sequencing data were extracted from the analysis of tumor tissues, which may introduce systematic bias at the cellular level. Therefore, in subsequent studies, we intend to analyze SLC35A2 using single-cell RNA sequencing. It needs further confirmation in the future to elucidate the underlying mechanism of SLC35A2 in cancer occurrence, progression, metastasis, and immunity. Altogether, our study indicated that the SLC35A2 could be a potential biomarker for clinical significance and immunotherapy response. Notably, the association between SLC35A2, the clinical significance, and the immunotherapy response requires further validation in future studies.

## 5 Conclusion

In summary, our comprehensive pan-cancer analysis of SLC35A2 helped to characterize SLC35A2 in multiple types of cancers. SLC35A2 overexpression predicts a worse prognosis in five types of cancer and is positively correlated with advanced clinical stages in 18 types of cancer. SLC35A2 expression significantly correlates with immune checkpoint genes and immune checkpoint therapy-related markers, including TMB, MSI, and TIDE. SLC35A2 may further affect tumor immunity mainly by regulating CD8+T cells, NK cells, B cells, and CD4+T cells, and the effect of SLC35A2 on immunity varies across tumor types. Furthermore, in four independent immunotherapy cohorts that included patients with LUAD, LUSC, SKCM, and BLCA, SLC35A2 had a good predictive effect on immunotherapy response. We found that targeted therapy against SLC35A2 may be helpful for patients with metastatic castration-resistant cancer. To date, the comprehensively analyze SLC35A2 in pan-cancer has not yet been reported. This study highlighted that SLC35A2 may serve as a biomarker for predicting the prognosis of pan-cancer and the response to immunotherapy.

## Data availability statement

The original contributions presented in the study are included in the article/**Supplementary Material**. Further inquiries can be directed to the corresponding authors.

## Ethics statement

The studies involving human participants were reviewed and approved by The Clinical Research Ethics Committee of Jiangmen Central Hospital (Approval number: 2022-098). The patients/participants provided their written informed consent to participate in this study.

## Author contributions

SX, XC, JF, and HC performed the data analysis and interpreted the results. SX, ZL, XC, and YL prepared the draft. All authors contributed to the article and approved the submitted version.

## Supplementary material

Supplementary Figure 1 Comparison of solute carrier family 35 member A2 (SLC35A2) methylation levels between the tumor and normal groups. Differences between means were determined by Student’s *t*-test. *Indicates p < 0.05.

Supplementary Figure 2 Prognostic differences in solute carrier family 35 member A2 (SLC35A2) at high- and low- methylation levels compared by Kaplan–Meier survival analysis. Differences were determined by the Log-rank test. p < 0.05 indicates significance.

Supplementary Figure 3 UGT1A4, UGT1A3, and UGT1A7 expression in samples with wild-type or mutated solute carrier family 35 member A2 (SLC35A2) in colon adenocarcinoma (COAD), lung squamous cell carcinoma (LUSC), and skin cutaneous melanoma (SKCM), respectively. Differences between means were determined by Student’s *t*-test. *Indicates P < 0.05.

Supplementary Figure 4 Solute carrier family 35 member A2 (SLC35A2) expression in different tissues. SLC35A2 expression in different healthy **(A)** tissues and **(B)** cell types.

Supplementary Figure 5 Kaplan–Meier survival curves comparing the high and low expression of the solute carrier family 35 member A2 (SLC35A2) gene in 19 independent datasets.

Supplementary Figure 6 Pan-cancer solute carrier family 35 member A2 (SLC35A2) expression in different cancer stages as defined by World Health Organization. Differences between means were determined by Student’s *t*-test, and the P value was indicated.

Supplementary Figure 7 Solute carrier family 35 member A2 (SLC35A2)-related gene enrichment and pathway analysis. **(A)** A heatmap representation of the relevant SLC35A2 and gene set variation analysis (GSVA) scores of functional pathways. **(B)** STRING protein network map of SLC35A2-binding proteins. **(C)** Presentation of hub genes through Cytoscape software.

Supplementary Figure 8 Association between SLC35A2 expression and the immune and stromal scores.

Supplementary Figure 9 Spearman correlation heatmap showing the relationship between solute carrier family 35 member A2 (SLC35A2) expression and **(A)** chemokine, chemokine receptors, immunomodulators, and MHC and **(B)** checkpoints across human cancers.

Supplementary Figure 10 Different algorithms for immuno-infiltration analysis. Spearman correlation heatmap showing the relationship between solute carrier family 35 member A2 (SLC35A2) expression and tumor-infiltrating lymphocytes (TILs) based on different algorithms. **(A)** CIBERSORT-ABS, **(B)** CIBERSORT, **(C)** quanTIseq, **(D)** MCP-counter, **(E)** EPIC, **(F)** TIMER, and **(G)** xCell.

Supplementary Figure 11 Molecular docking. **(A, B)** 2D and 3D diagrams showing the molecular docking of CYP17 to abiraterone.
