# Supplementary figures and images for: Comprehensive analysis of 33 human cancers reveals clinical implications and immunotherapeutic value of the solute carrier family 35 member A2
Source: Front Immunol. 2023 May 18;14:1155182. doi: 10.3389/fimmu.2023.1155182 (PMC10232969; doi:10.3389/fimmu.2023.1155182)

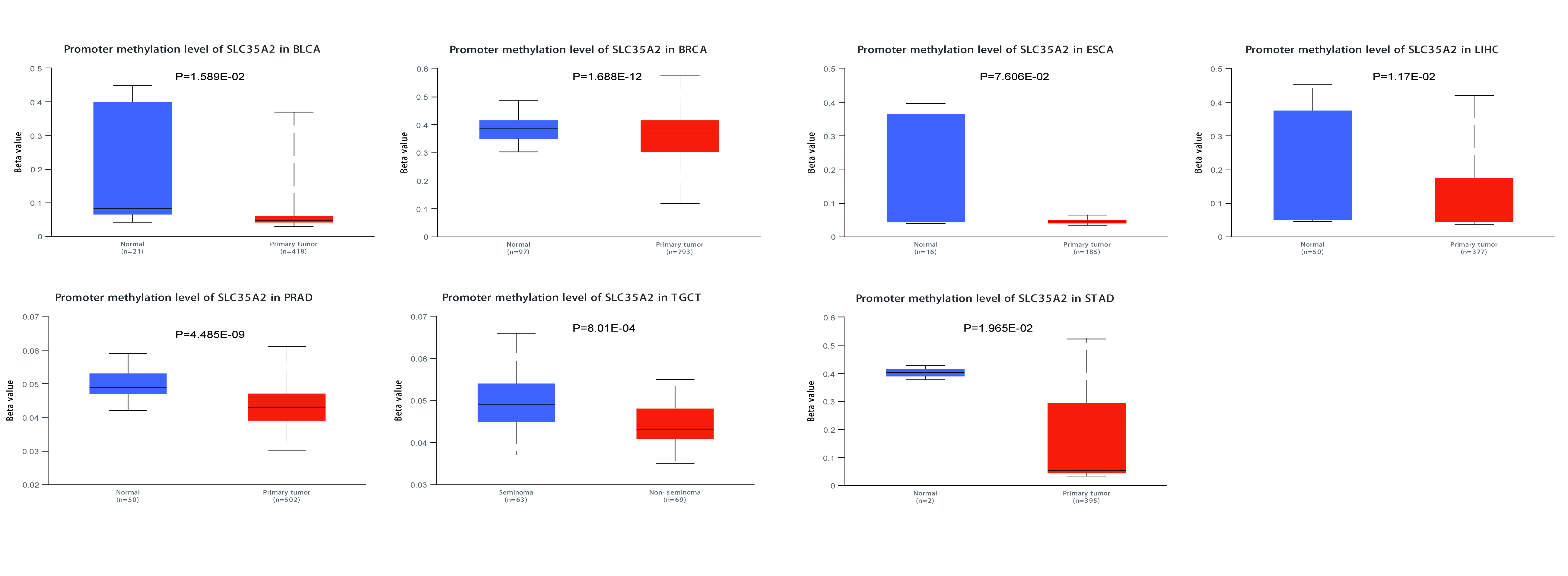

Supplement: Supplementary Figure 1 — Comparison of solute carrier family 35 member A2 (SLC35A2) methylation levels between the tumor and normal groups. Differences between means were determined by Student’s t-test. *Indicates p < 0.05. [file Image_1.tif]

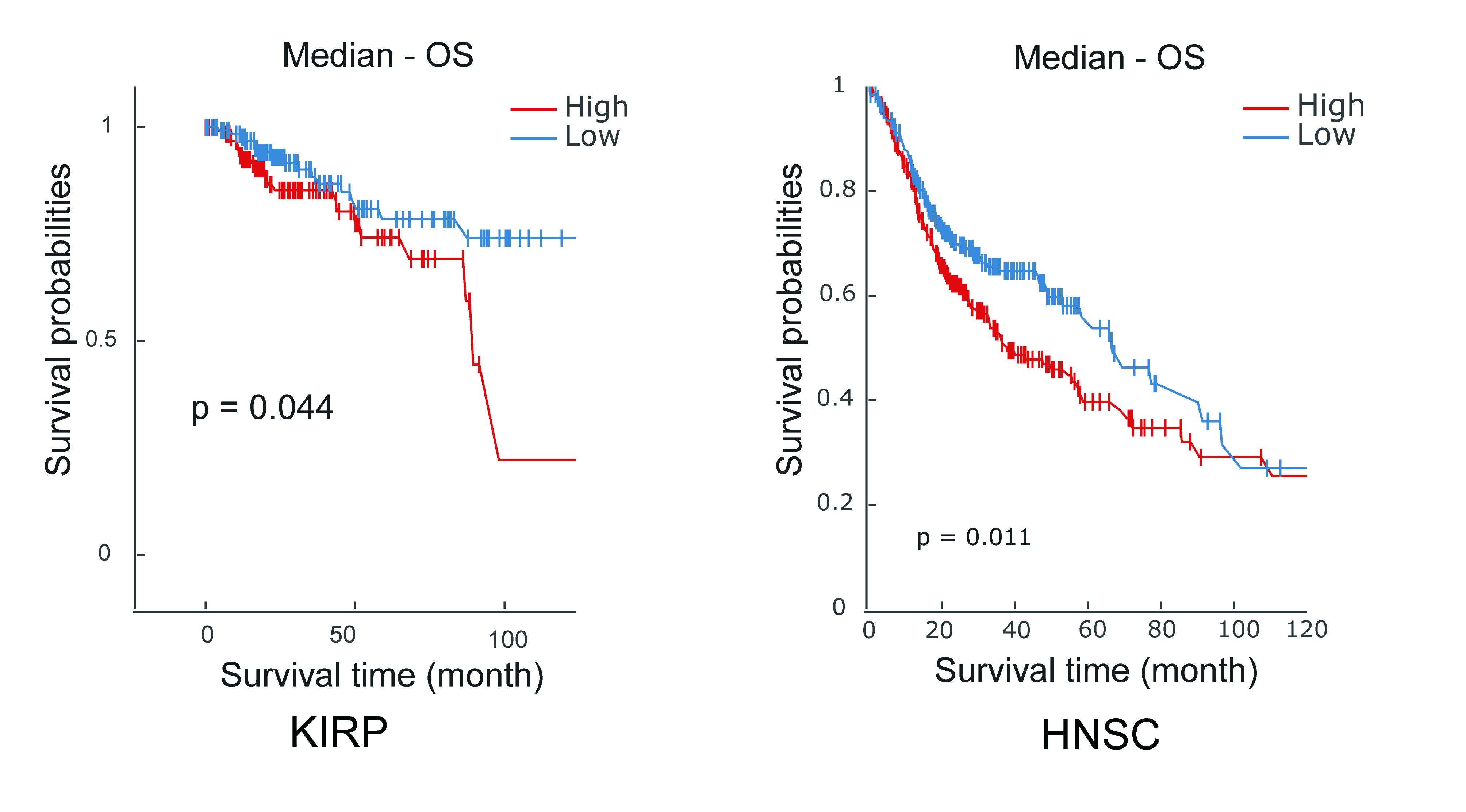

Supplement: Supplementary Figure 2 — Prognostic differences in solute carrier family 35 member A2 (SLC35A2) at high- and low- methylation levels compared by Kaplan–Meier survival analysis. Differences were determined by the Log-rank test. p < 0.05 indicates significance. [file Image_2.tif]

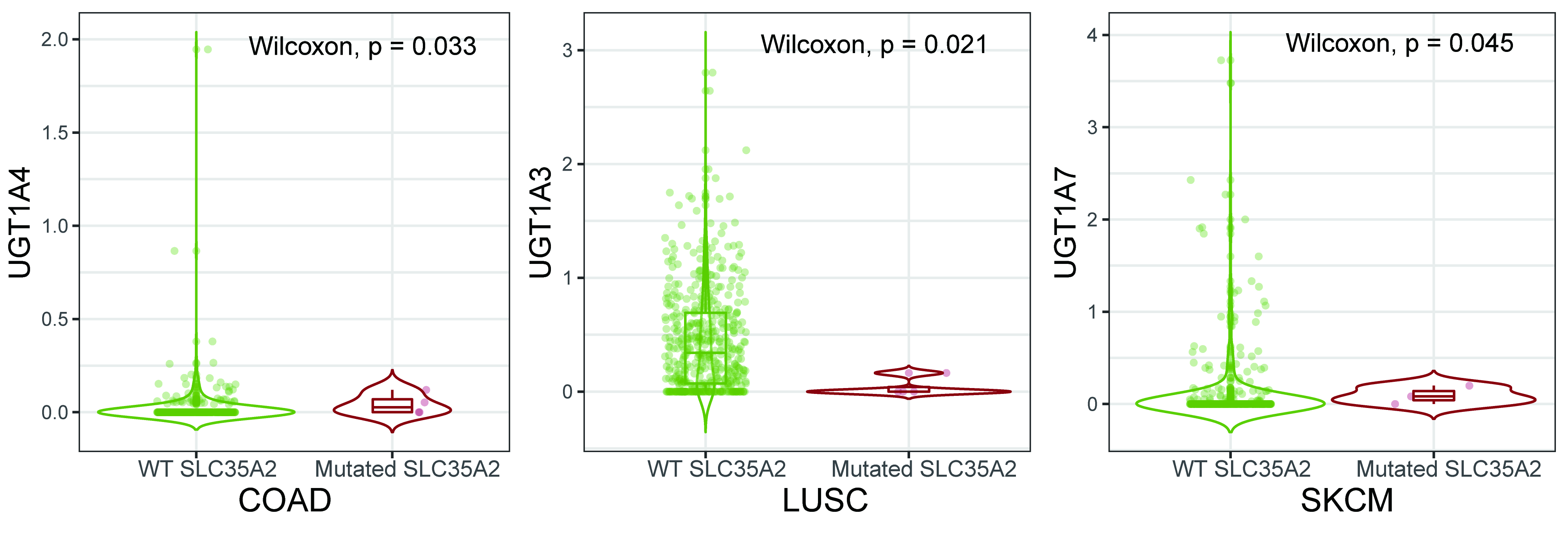

Supplement: Supplementary Figure 3 — UGT1A4, UGT1A3, and UGT1A7 expression in samples with wild-type or mutated solute carrier family 35 member A2 (SLC35A2) in colon adenocarcinoma (COAD), lung squamous cell carcinoma (LUSC), and skin cutaneous melanoma (SKCM), respectively. Differences between means were determined by Student’s t-test. *Indicates P < 0.05. [file Image_3.tif]

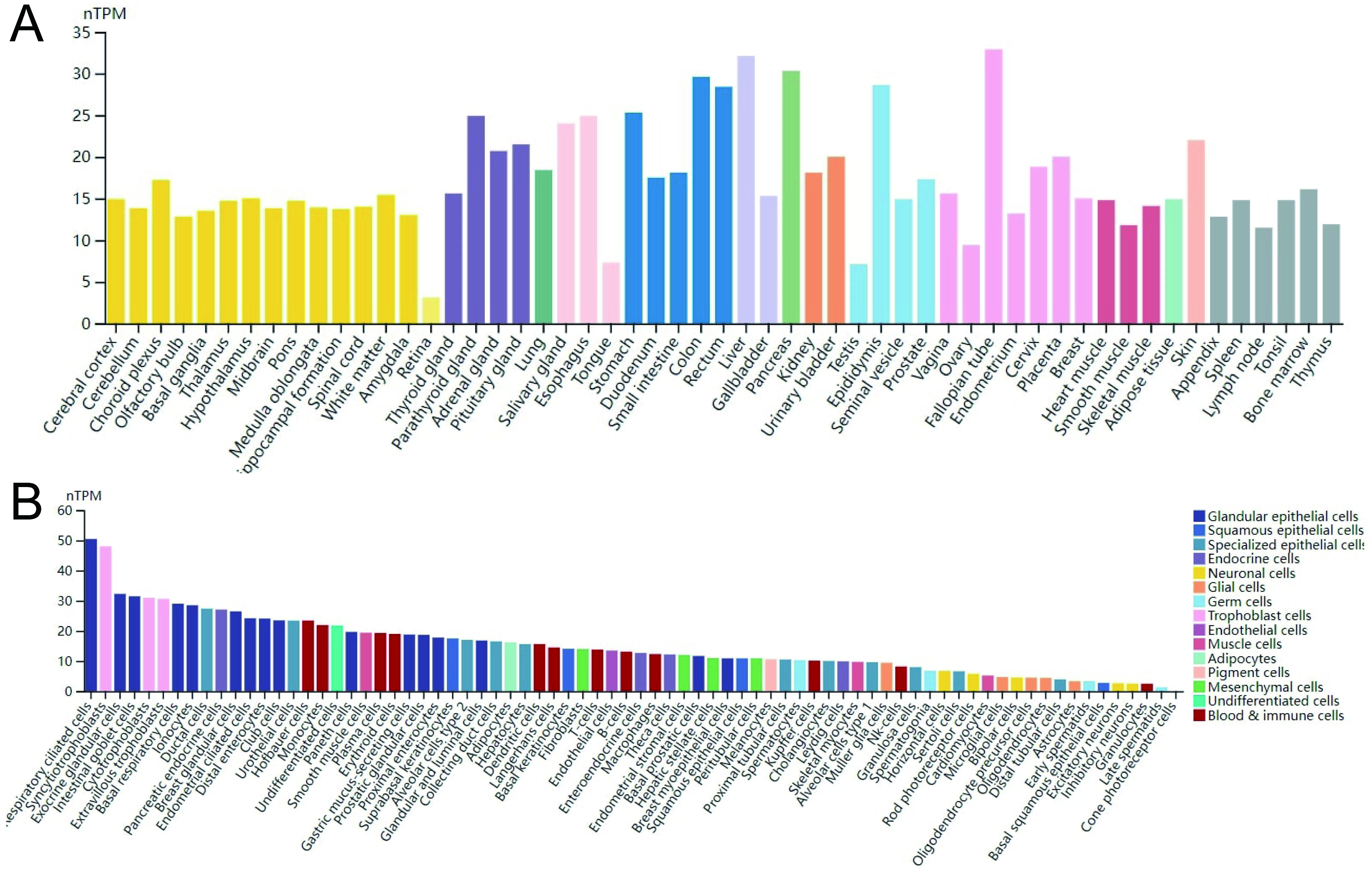

Supplement: Supplementary Figure 4 — Solute carrier family 35 member A2 (SLC35A2) expression in different tissues. SLC35A2 expression in different healthy (A) tissues and (B) cell types. [file Image_4.tif]

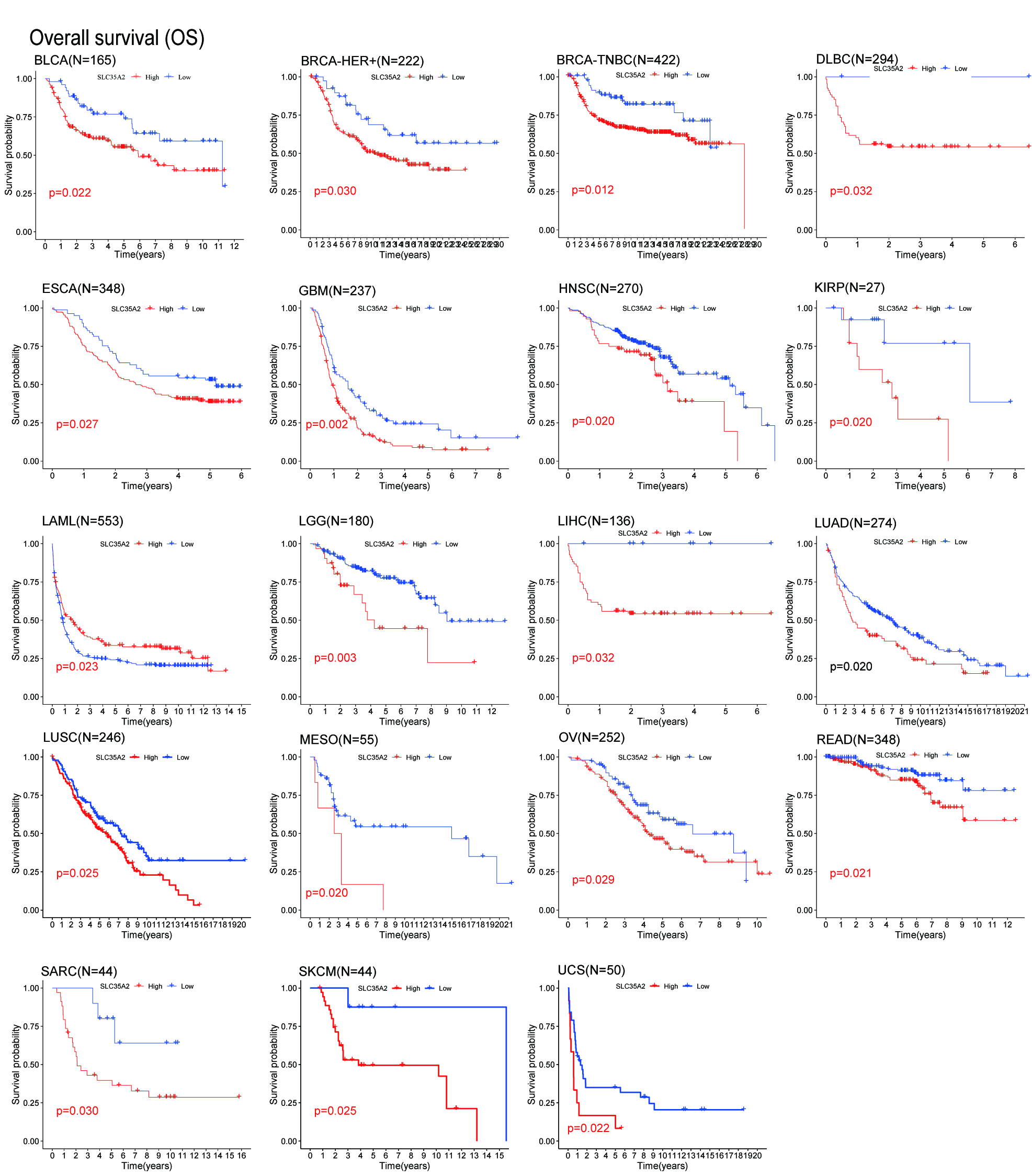

Supplement: Supplementary Figure 5 — Kaplan–Meier survival curves comparing the high and low expression of the solute carrier family 35 member A2 (SLC35A2) gene in 19 independent datasets. [file Image_5.tif]

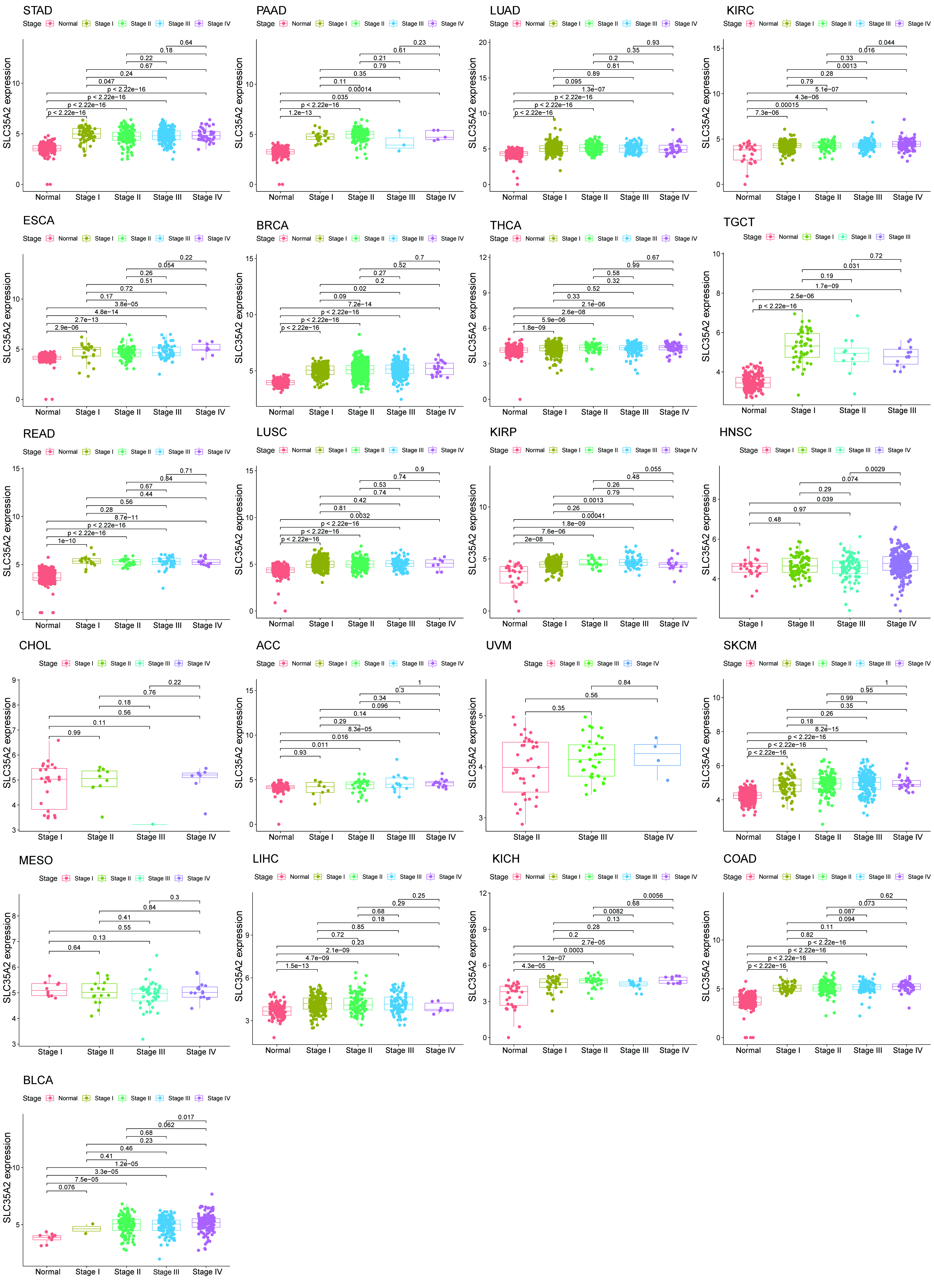

Supplement: Supplementary Figure 6 — Pan-cancer solute carrier family 35 member A2 (SLC35A2) expression in different cancer stages as defined by World Health Organization. Differences between means were determined by Student’s t-test, and the P value was indicated. [file Image_6.tif]

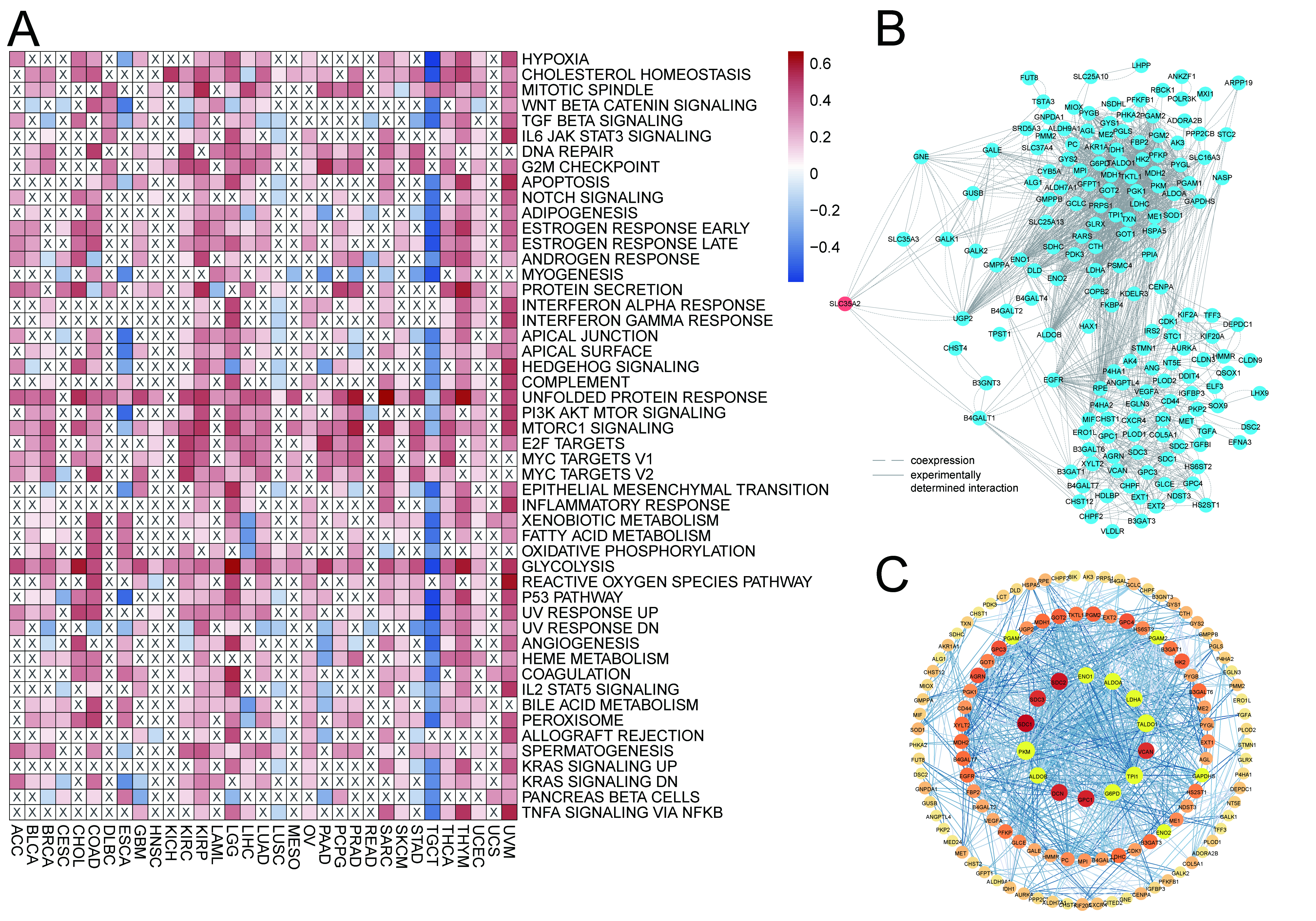

Supplement: Supplementary Figure 7 — Solute carrier family 35 member A2 (SLC35A2)-related gene enrichment and pathway analysis. (A) A heatmap representation of the relevant SLC35A2 and gene set variation analysis (GSVA) scores of functional pathways. (B) STRING protein network map of SLC35A2-binding proteins. (C) Presentation of hub genes through Cytoscape software. [file Image_7.tif]

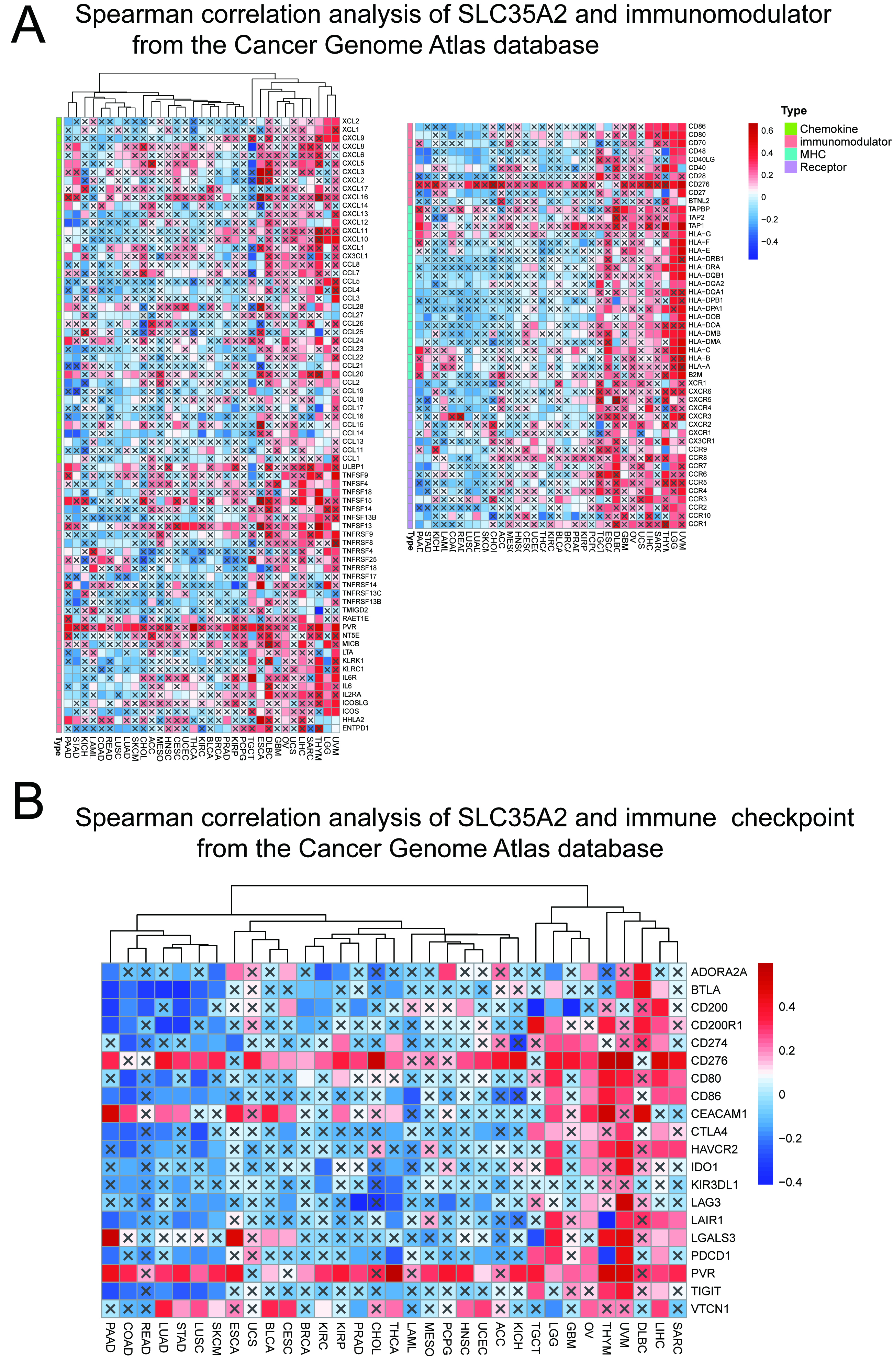

Supplement: Supplementary Figure 9 — Spearman correlation heatmap showing the relationship between solute carrier family 35 member A2 (SLC35A2) expression and (A) chemokine, chemokine receptors, immunomodulators, and MHC and (B) checkpoints across human cancers. [file Image_9.tif]

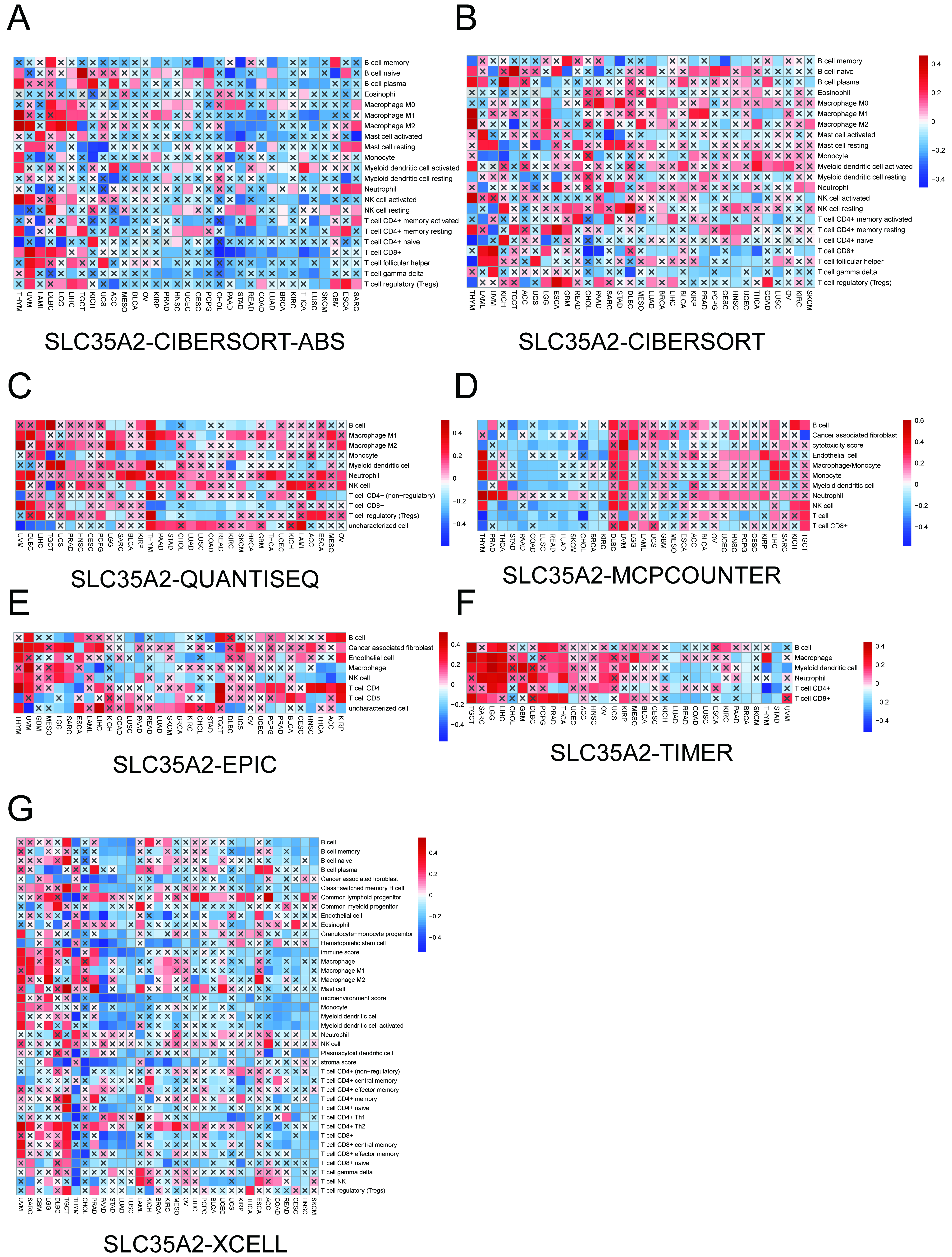

Supplement: Supplementary Figure 10 — Different algorithms for immuno-infiltration analysis. Spearman correlation heatmap showing the relationship between solute carrier family 35 member A2 (SLC35A2) expression and tumor-infiltrating lymphocytes (TILs) based on different algorithms. (A) CIBERSORT-ABS, (B) CIBERSORT, (C) quanTIseq, (D) MCP-counter, (E) EPIC, (F) TIMER, and (G) xCell. [file Image_10.tif]

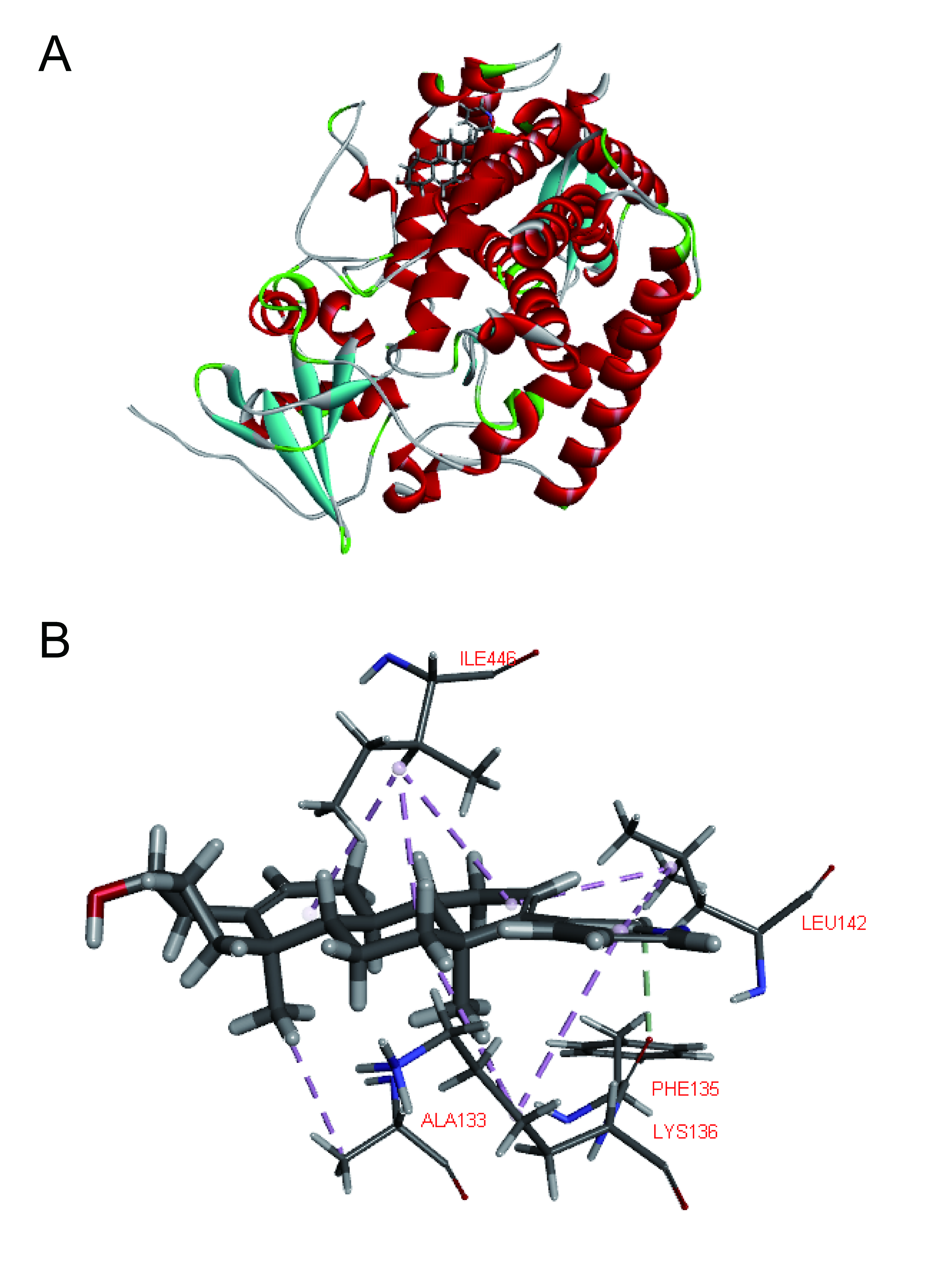

Supplement: Supplementary Figure 11 — Molecular docking. (A, B) 2D and 3D diagrams showing the molecular docking of CYP17 to abiraterone. [file Image_11.tif]
